# siRNAs regulate DNA methylation and interfere with gene and lncRNA expression in the heterozygous polyploid switchgrass

**DOI:** 10.1186/s13068-018-1202-0

**Published:** 2018-07-24

**Authors:** Haidong Yan, Aureliano Bombarely, Bin Xu, Taylor P. Frazier, Chengran Wang, Peilin Chen, Jing Chen, Tomas Hasing, Chenming Cui, Xinquan Zhang, Bingyu Zhao, Linkai Huang

**Affiliations:** 1 Department of Grassland Science, Animal Science and Technology College, Sichuan Agricultural University, Chengdu, 611130 China; 2 Department of Horticulture, Virginia Tech, Blacksburg, VA 24061 USA; 3 College of Grassland Science, Nanjing Agricultural University, Nanjing, 210095 China; 4 Department of Plant Sciences, University of Tennessee, Knoxville, TN 37996 USA; 5 Department of Plant Pathology, Physiology, and Weed Science, Virginia Tech, Blacksburg, VA 24061 USA

**Keywords:** *Panicum virgatum* L., DNA methylation, Gene expression, Non-coding RNAs, RNA-dependent DNA methylation, Differentially methylated regions

## Abstract

**Background:**

Understanding the DNA methylome and its relationship with non-coding RNAs, including microRNAs (miRNAs) and long non-coding RNAs (lncRNAs), is essential for elucidating the molecular mechanisms underlying key biological processes in plants. Few studies have examined the functional roles of the DNA methylome in grass species with highly heterozygous polyploid genomes.

**Results:**

We performed genome-wide DNA methylation profiling in the tetraploid switchgrass (*Panicum virgatum* L.) cultivar ‘Alamo’ using bisulfite sequencing. Single-base-resolution methylation patterns were observed in switchgrass leaf and root tissues, which allowed for characterization of the relationship between DNA methylation and mRNA, miRNA, and lncRNA populations. The results of this study revealed that siRNAs positively regulate DNA methylation of the mCHH sites surrounding genes, and that DNA methylation interferes with gene and lncRNA expression in switchgrass. Ninety-six genes covered by differentially methylated regions (DMRs) were annotated by GO analysis as being involved in stimulus-related processes. Functionally, 82% (79/96) of these genes were found to be hypomethylated in switchgrass root tissue. Sequencing analysis of lncRNAs identified two lncRNAs that are potential precursors of miRNAs, which are predicted to target genes that function in cellulose biosynthesis, stress regulation, and stem and root development.

**Conclusions:**

This study characterized the DNA methylome in switchgrass and elucidated its relevance to gene and non-coding RNAs. These results provide valuable genomic resources and references that will aid further epigenetic research in this important biofuel crop.

**Electronic supplementary material:**

The online version of this article (10.1186/s13068-018-1202-0) contains supplementary material, which is available to authorized users.

## Background

Switchgrass (*Panicum virgatum* L.) is a warm season, perennial, C_4_ grass native to the North American prairie [1]. Switchgrass has been identified as one of the most promising feedstock grass species for biofuel production [2]. There are two different ecotypes of switchgrass, upland and lowland, which are distinguished according to their natural habitats. The majority of upland ecotypes are octoploid (2n = 8x = 72) and most lowland ecotypes are tetraploid (2n = 4x = 36) [3, 4]. The tetraploid switchgrass genome is comprised of two sets of closely related homeologous chromosomes, and it is highly heterozygous due to the outcrossing nature of the species [5, 6].

DNA methylation is an epigenetic mechanism that influences molecular processes pertaining to plant growth and development [7], including immunity to virus infection [8], gene imprinting [9], transposon silencing [10, 11], and embryogenesis [12]. Methylation on the cytosine of three different nucleotide patterns is common in plants: CG, CHG, and CHH (H = A, T, or C) contexts [13]. DNA methylation can induce repression or activation of genes and transposable elements (TEs) in response to environmental and developmental signals. Therefore, characterization of cytosine methylation patterns can provide valuable information that allows for a greater understanding of the regulation of gene expression, and the dynamics of TEs in the shaping of the plant genome architecture.

Non-coding RNAs are RNA molecules that are not translated into proteins. There are two major classes of non-coding RNAs: microRNAs (miRNAs) and long non-coding RNAs (lncRNAs). MiRNAs are small sequences between 20 and 24 nt in length. These molecules often regulate gene expression post-transcriptionally by either mRNA cleavage or translation repression [14, 15]. LncRNAs are transcripts of more than 200 nucleotides that have numerous functions within the plant cell. They have been shown to target promoters upstream of genes, function as precursors of small RNAs, generate alternatively spliced transcripts, produce endogenous small interfering RNAs, and form RNA–protein complexes [16]. Non-coding RNAs have been shown to be closely related to DNA methylation. It has been reported that siRNAs can increase CHH methylation in many plant species through the RNA-dependent DNA methylation (RdDM) pathway [17–20]. More recently, lncRNAs have also been demonstrated to have key roles in the regulation of DNA methylation in mammals [21, 22]. For example, the long non-coding ecCEBPA transcript, encoded within the CEBPA gene, binds to DNMT1 and limits methylation of the CEBPA gene [21]. Alternatively, the H19 lncRNA alters genome-wide DNA methylation patterns by regulating S-adenosylhomocysteine hydrolase in mammals [22]. To date, the relationship between lncRNAs and DNA methylation in plants, especially in C4 grasses, has not been extensively studied [23, 24].

C4 grasses are the most prominent in the grass family and include some of the most economically important crop species such as maize (*Zea mays*), great millet (*sorghum bicolor*), sugarcane (*Saccharum officinarum*), and switchgrass. Primary production of these crops annually accounts for roughly 25% of arable land [25]. Currently, DNA methylation patterns have been characterized in several C3 grasses such as rice (*Oryza sativa*) [26], *Brachypodium distachyon* [27], and wheat [28]; however, few DNA methylation studies have been performed on C4 grasses, such as switchgrass, which usually contain highly heterozygous and polyploid genomes. Thus far, DNA methylation studies in model plants, such as *Arabidopsis thaliana*, rice, and *B. distachyon*, have generated several conserved rules: (1) mCG is dominating among the three methylated contexts; (2) methylation may act as a mechanism for controlling gene expression, but the methylation variation depends on types of contexts and gene region; and (3) RdDM pathways are highly associated with mCHH [17, 26, 27, 29, 30]. Since switchgrass is a polyploid, it would be interesting to investigate if it has unique methylation patterns that are different from the general rules identified in other model plant species.

In this study, we performed global DNA methylation sequencing on leaf and root tissues of tetraploid switchgrass (cv. Alamo) and profiled expression of mRNAs, miRNAs, and lncRNAs in the leaf tissue. Our results revealed that siRNAs positively regulated DNA methylation at the mCHH sites surrounding genes and that DNA methylation may interfere with both gene and lncRNA expression in the polyploid switchgrass genome. In addition, we identified a small subset of genes hypomethylated in the root tissue that were characterized by differentially methylated regions (DMRs), which could potentially be involved in stimulus-related GO processes. Finally, we predicted two precursors (lncRNAs) of miRNAs that might function in cellulose biosynthesis, stress regulation, and stem and root development. Overall, this study described the DNA methylome of switchgrass and its relation to gene and non-coding RNAs. These results provide a platform for future epigenetic studies in biofuel crops.

## Results

### Single-base resolution landscapes of DNA methylation in switchgrass

Whole-genome bisulfite sequencing was applied to genomic DNA of switchgrass extracted from leaves and roots. In total, 411 million reads were generated for switchgrass leaf tissue and 392 million reads were generated for switchgrass root tissue. Of the reads generated in this study, 334 (81%) million reads from the leaf sample and 304 (77%) million reads from the root sample could be aligned to the reference genome of switchgrass (V4.1) (Additional file 1: Table S1). For both tissues, the effective coverage rate of cytosine was analyzed at the chromosome level, as well as in genic and repetitive regions, to evaluate the quality of the methylation data (Additional file 2: Data S1). The effective coverage rates of chromosomes ranged between 75 and 85%, whereas the coverage rates of gene regions ranged between 82 and 87%. For the repetitive regions, low complexity repeats (cryptic, tandem, and interspersed repeats) had coverage rates between 56 and 64%, and the remaining repetitive elements had coverage rates ranging from 69 to 86% (Additional file 2: Data S1). In addition, the sequencing depths of the leaf and root samples were calculated to be 29X and 26X, respectively. These numbers suggest that the amount of detected cytosines reached saturation (Additional file 1: Table S1 and Additional file 3: Figure S1). Overall, the methylation data generated in this experiment had a highly effective coverage rate (~ 80%) of cytosine. The sequencing depths indicated a sufficient level for further methylation analysis.

**Fig. 1:**
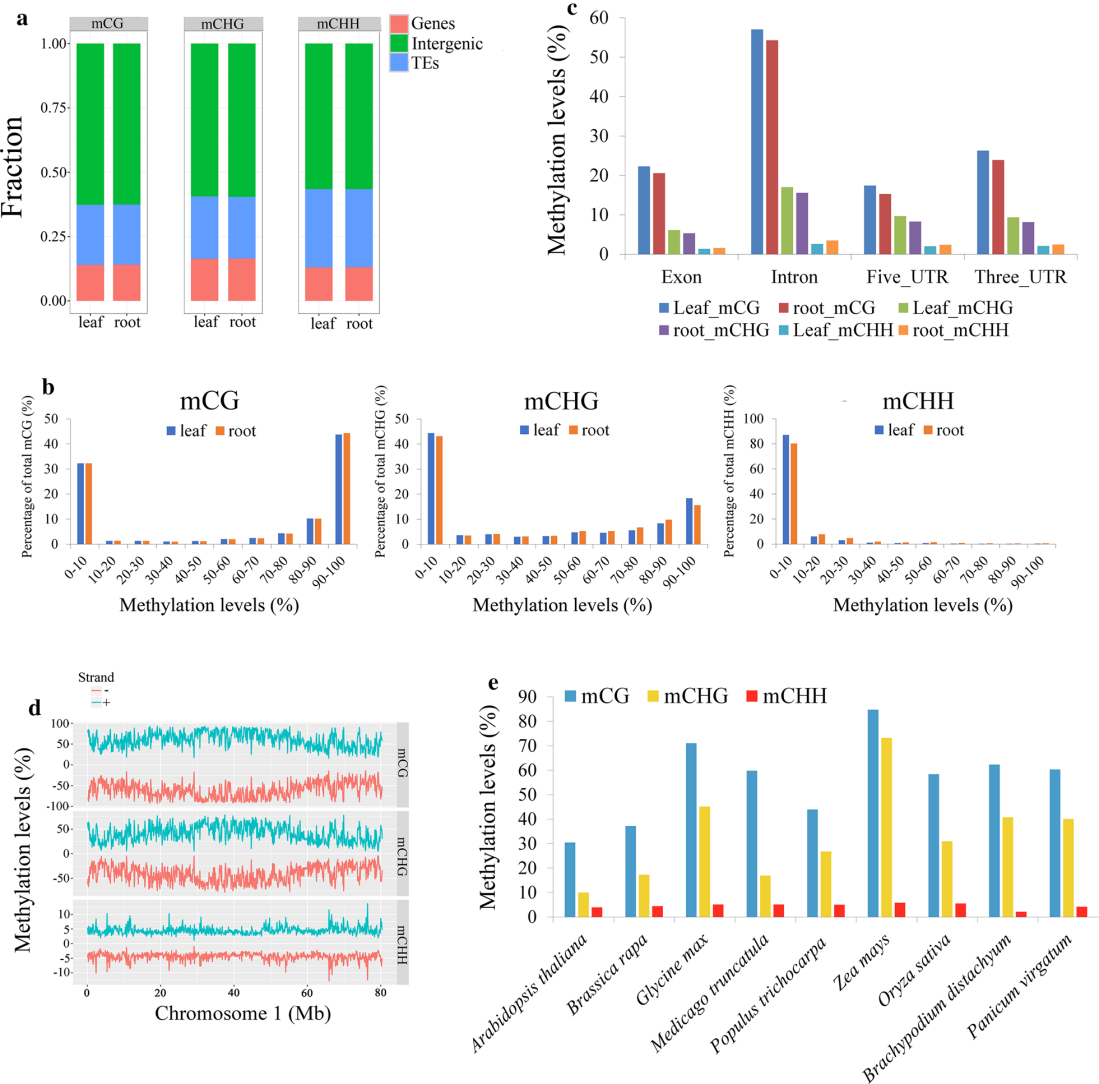
DNA methylation levels of three methylated contexts (mCG, mCHG, and mCHH) and distribution in the leaf and root tissues of switchgrass. **a** The percentage of methylated cytosine sites distribution of mCG, mCHG, mCHH in genes, intergenic regions, and TEs. **b** Distribution of the methylation levels in each methylated context (the *x*-axis indicates the methylation level is divided into ten bins from 0 to 100%, and the *y*-axis represents methylation levels in each bin). **c** Methylation level in different parts of gene region (Exon; Intron; Five_UTR; Three_UTR). **d** Methylation level in 80-kb windows throughout chromosome 1a in the leaf tissue of switchgrass. The red line means ‘−’ strand, and the blue line means ‘+’ strand. **e** The methylation levels of nine different species shown in Additional file 5: Table S2

A total of 66 and 77 million methylcytosines (mCs) were identified in the genomic DNA isolated from switchgrass leaves and roots, respectively. For the leaf tissue, the cytosine methylation levels were higher at the mCG sites (60%) than those at mCHG (40%) and mCHH (4%) sites (Table 1). The data from the root tissue displayed similar patterns of mCG (59%) and mCHG sites (39%), and increased mCHH sites (7%) (Table 1). Most of the mCs identified in this study were located in unannotated intergenic and TE regions (Fig. 1a). Most mCG and mCHG sites were either highly methylated (methylation levels > 80%) or lowly methylated (methylation levels < 10%). These sites shared similar patterns in both tissues. Most mCHH sites, however, only displayed low methylation levels (< 15%) (Fig. 1b). Interestingly, the methylation levels of the intronic regions were higher than the levels of other regions (Fig. 1c).

**Table 1:** The number of epigenetically modified sites and the estimated proportion of the genome methylated according to three methylation contexts in leaf and root tissues of switchgrass

	mCG	mCHG	mCHH
Leaf			
Number of modified sites	29,369,883	23,637,970	13,739,344
Proportion of the genome (%)	44.00	35.41	20.58
Methylation level (%)	60.34	40.06	4.16
Root			
Number of modified sites	30,097,757	23,937,098	22,509,824
Proportion of the genome (%)	39.32	31.27	29.41
Methylation level (%)	59.35	38.95	6.67

A chromosome-scale view of the methylation sites and their levels was generated from the sequencing data (Fig. 1d and Additional file 4: Figure S2). It was discovered that the terminal regions of the chromosomes had a lower level of methylation at mCG and mCHG sites while methylation at mCHH sites exhibited a more flat distribution pattern.

Methylation across the three types was also found to vary for different plant species (Fig. 1e and Additional file 5: Table S2). For each species analyzed, mCG and mCHG sites had higher methylation levels across the genomes than mCHH sites, which were universally the lowest (< 6%). Specifically, maize and *Arabidopsis* had the highest and lowest proportion of mCG (85%) and mCHG (10%), respectively. For the four Poaceae plants examined in this study, the levels of mCG and mCHG were higher in switchgrass than *B. distachyon* and rice, but lower than similar sites in maize (Fig. 1e).

### DNA methylation profiles in genic and transposable element rich regions

**Fig. 2:**
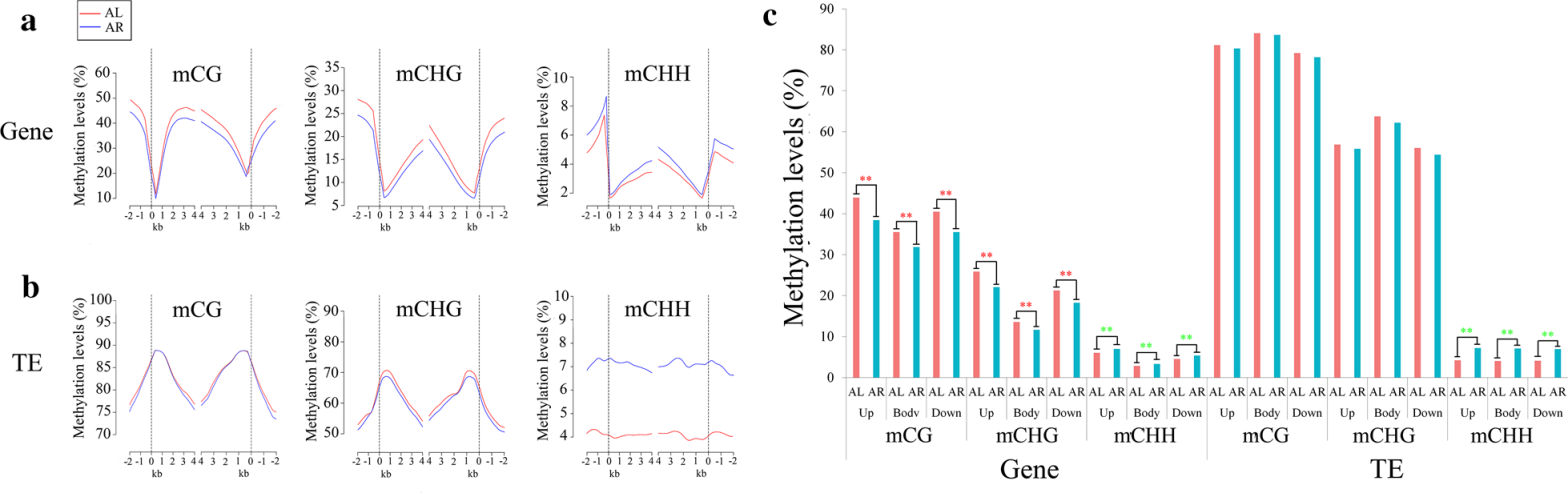
DNA methylation profiles and levels of gene and TE regions in leaf and root tissues of switchgrass. **a** DNA methylation patterns in gene regions. **b** DNA methylation patterns in TE regions. The average methylation level for each 100-bp interval is plotted. The dashed lines for gene and TE regions indicate the transcriptional start (left) and end (right) sites. AL and AR indicate leaf and root tissues, respectively, in switchgrass. **c** Comparison of DNA methylation levels between leaf and root tissues of switchgrass in gene and TE body and their flanking regions (2-kb), respectively. * means the *p* < 0.05. ** means the *p* < 0.01

Methylation patterns in the protein-coding genes were examined by analyzing the three common methylation contexts across the gene body regions and the 2-kb regions flanking the genes (Fig. 2a, b). Relatively high methylation levels were discovered on the CG sites, followed by the CHG and CHH sites in the genic regions (Fig. 2a). Interestingly, the 5′ flanking region upstream of the genes exhibited low levels of methylation that increased at the mCG and mCHG sites near the start of the coding sequence but subsequently decreased across the gene body. Another region of highly methylated region for these mCG and mCHG sites was observed near the point of transcription termination; however, lower levels of methylation were then found in the 3′ flanking region (Fig. 2a, c).

The methylation patterns of TEs were also examined similarly to the genic regions and included both up- and downstream regions (Fig. 2b). The average methylation level of TEs was higher than that of protein-coding genes (Fig. 2a–c). The mCG and mCHG sites showed a similar trend where the methylation levels were much higher at the start and end point of transcription than in both the TE gene body and flanking regions. The methylation levels of mCHH sites were similar at the start and end points, as well as across the body and flanking regions, for all examined TEs (Fig. 2b). Previous reports have suggested that methylation levels of CG and CHG are positively correlated with genome size in plants [17], which was re-confirmed in this study using different set of plant genomes including switchgrass (Additional file 6: Figure S3). In addition, it has been shown that proliferation of TEs contributes to the genome size [31]. Several reports, along with the data generated in this study, have discovered that the average methylation levels in TEs are higher than in the genic regions (Fig. 2a–c) [19, 20, 32]. Therefore, TE methylation levels contribute to the overall genome methylation levels in plants and regulation of these levels may positively correlate with plant genome sizes.

**Fig. 3:**
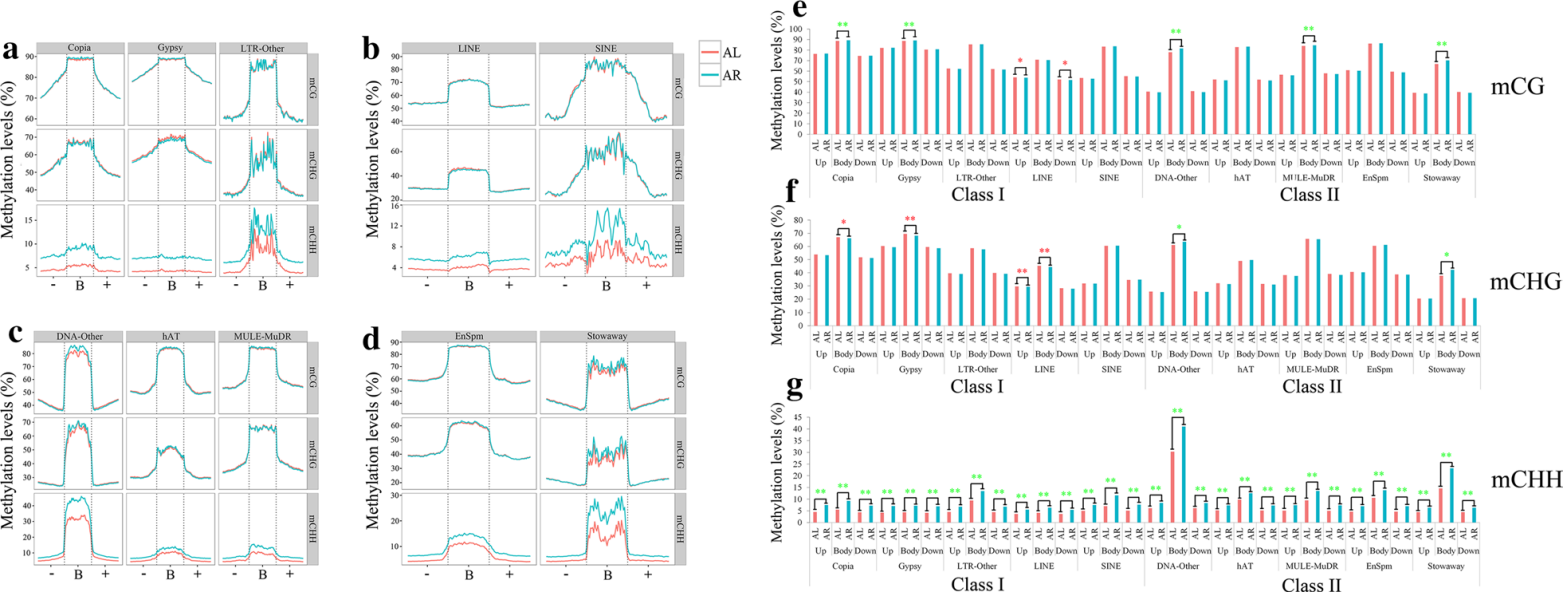
DNA methylation profiles and levels of class I and class II TEs in leaf and root tissues of switchgrass. **a**, **b** Average methylation level distribution over class I TEs (Retrotransposons; Copia, Gypsy, LTR-Other, LINE, and SINE). **c**, **d** Average methylation level distribution over class II TEs (DNA transposons; DNA transposons including DNA-Other, hAT, MULE-MuDR, EnSpm, and Stowaway). “−”, B” and “+” mean upstream (2-kb), body and downstream (2-kb) regions of TEs, respectively. AL and AR indicate leaf and root tissues, respectively, in switchgrass. **e**–**g** comparison of DNA methylation levels in Class I and Class II TEs between leaf and root tissues of switchgrass in the body and 2 kb-flanking regions. * means the *p* < 0.05, and ** means the *p* < 0.01. The red asterisk means hyper-methylation in the leaf tissue, and the green asterisk means hyper-methylation in the root tissue

To further characterize the methylation patterns within TEs, the methylation trends of ten major families of TEs were compared. These TEs were grouped into two classes: class I (retrotransposons including Copia, Gypsy, LTR-Other, LINE, and SINE), and class II (DNA transposons including DNA-Other, hAT, MULE-MuDR, EnSpm, and Stowaway) (Fig. 3a–d). While the methylation distributions of the ten TE families were different, they all displayed a similar pattern with obviously higher methylation levels in body regions than flanking regions. For the class I TEs, significantly higher methylation levels were detected on mCG and mCHG sites than on mCHH sites (Fig. 3a, b). In addition, the mCG and mCHG positions were hyper-methylated in the body regions of all class I TEs, while the mCHH levels were equally methylated to those in the up- and downstream regions in Copia, Gypsy, and LINE. The mCHH sites were found to be hyper-methylated in the body regions of Copia and Gypsy. For the class II TEs, the levels of mCG and mCHG were also higher than mCHH (Fig. 3c, d). In comparison with the class I TEs, the class II TEs methylation patterns showed a significant increase in methylation levels at all three methylation contexts across the DNA transposon gene body rather than in the flanking regions, despite mCHH being slightly elevated in the body regions of hAT and MULE-MuDR (Fig. 3c, d). The levels of mCG and mCHG were overall higher in class I than class II TEs (Additional file 7: Figure S4; Additional file 8: Table S3). Transposition of non-LTR retrotransposons (LINE and SINE) is rarely observed in plants, indicating that these retroelements are inactive [33]. This could be attributed to the high methylation levels of mCG and mCHG sites observed in both LINE and SINE (Fig. 3b).

### DNA methylation differences between leaf and root tissues in switchgrass

To identify global differentially methylated DNA between leaf and root tissues, the methylation levels of both genic and TE regions were compared. The analysis of these two genomic components was further divided into upstream, body, and downstream regions (Fig. 2c; Additional file 9: Table S4). In the genic regions, the mCG and mCHG levels were significantly higher in leaves compared to roots, whereas the mCHH levels were significantly lower in leaves. In the TE regions, mCG and mCHG displayed no significant methylation differences between the leaf and root tissue. However, hypomethylated mCHH sites were observed in the TE body and flanking regions in the leaf tissue (Fig. 2c; Additional file 9: Table S4). Therefore, there is a tissue-specific regulation pattern of DNA methylation in the genic regions in switchgrass. These results are consistent with those found in *Arabidopsis*, Norway spruce (*Picea abies*), and castor bean (*Ricinus communis*) [17, 20, 34].

Differential methylation of the two TE classes was also observed between the leaf and root tissues in the TE body and flanking regions (Fig. 3e–g; Additional file 10: Table S5). For the mCG and mCHG sites in both TE classes, no significant methylation difference was identified between the leaf and root tissues (Fig. 2c); however, significant differences were detected when the methylated TEs were annotated into 15 regions (five regions per upstream, body, and downstream regions) for the two classes. For the class I TEs, four regions displayed significant mCG changes in leaves compared to roots, including two hypomethylated and two hyper-methylated regions. In addition, four regions were CHG hyper-methylated and all regions were CHH hypomethylated (Fig. 3e–g; Additional file 10: Table S5). For the class II TEs, three regions displayed significant CG hypomethylation and two regions were significantly CHG hypomethylated. All regions were CHH hypomethylated for this class in leaf tissue (Fig. 3e–g; Additional file 10: Table S5).

**Fig. 4:**
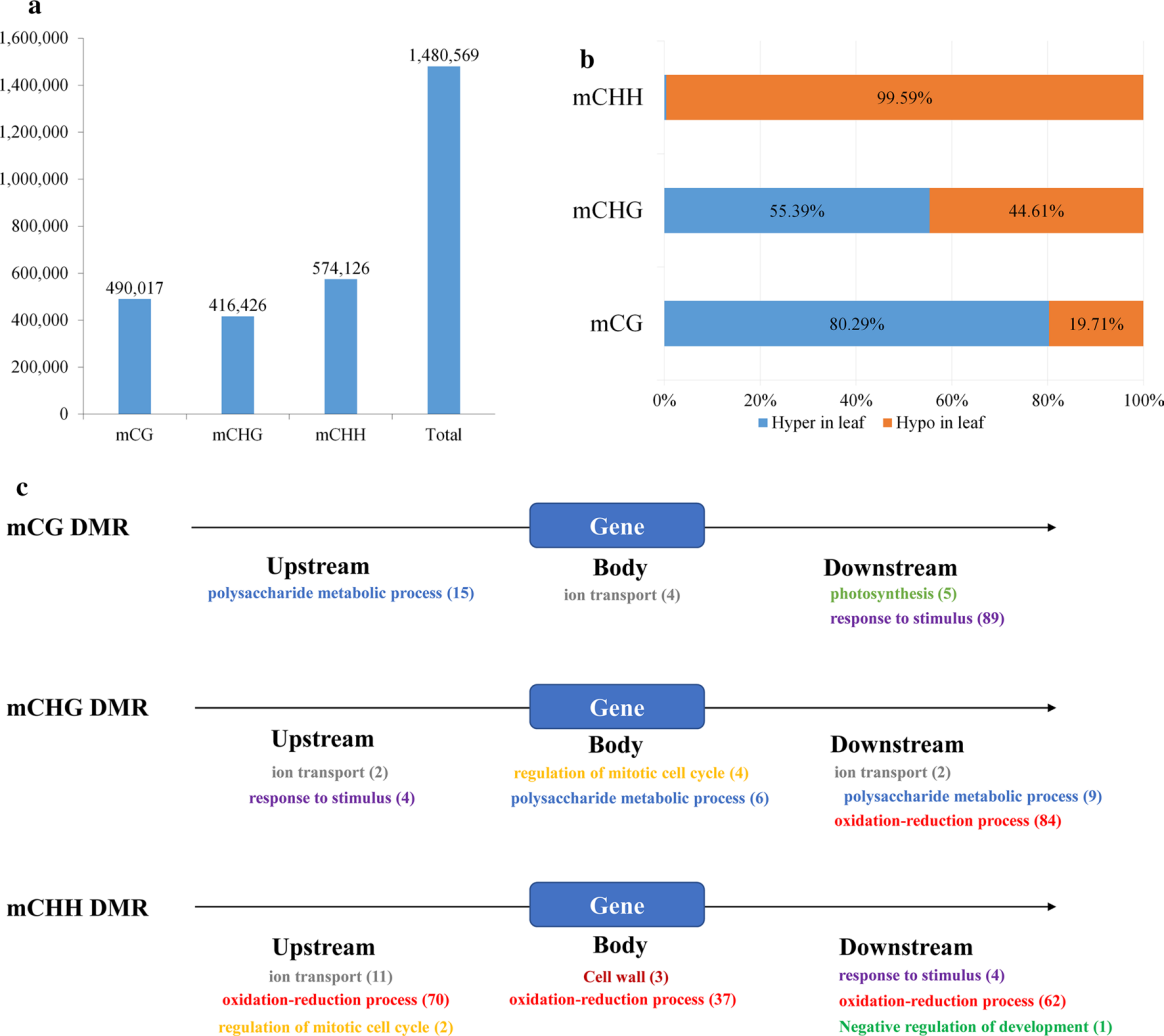
Different methylation regions between leaf and root tissues in switchgrass. **a** DMR numbers of three cytosine contexts. **b** Proportions of hyper- and hypo- DMRs in CG, CHG, and CHH contexts for the leaf tissue. **c** Biological processes for genes covered by DMRs in the flanking (2 kb) and gene body region in the GO annotation. Different color words and numbers showed the biological process and gene numbers, respectively. The same color represents the same biological process

Differential methylation regions (DMRs) play an important role for epigenetic methylation modifications across plant species [35]. In this study, the DMRs were analyzed for switchgrass through a sliding window by subtracting the leaf methylation loci from the root methylation loci. A total number of 1,480,569 DMRs were discovered, and the number of identified CHH DMRs (574,126) was larger than that for CG DMRs (490,017) and CHG DMRs (416,426) (Fig. 4a). Most mCG DMRs (80.29%) were hyper-methylated, and nearly half of mCHG DMRs (55.39%) were hyper-methylated in the leaf tissue. Surprisingly, almost all mCHH DMRs (99.59%) were hypomethylated in the leaf tissue (Fig. 4b).

Plant roots are essential for responding to abiotic stress stimuli and must quickly react to environmental changes by controlling key stress-related genes [36, 37]. Previous reports have showed that root and leaf tissues were differently methylated [38–41]. To detect differential methylation modifications between leaf and root tissues in switchgrass, we applied GO annotation towards genes covered by the DMRs (*p* < 0.05; Additional file 11: Data S2). The enriched molecular processes were different among all three methylation contexts and the locations of the DMRs within genes (Fig. 4c; Additional file 11: Data S2). We identified 96 genes covered by the DMRs that were involved in various response-related processes including response to stimulus (GO:0050896), response to abiotic stimulus (GO:0009628), regulation of response to stimulus (GO:0048583), response to temperature stimulus (GO:0009266), and response to cold and freezing (GO:0009409 and GO:0050826) (Additional file 12: Data S3; Additional file 11: Data S2; Fig. 4c). Eighty-nine genes were covered by downstream DMRs on the mCG sites and one gene was covered by mCHG and mCHH DMRs in the upstream and downstream regions, respectively. For the other six genes, three were covered by mCHG DMRs in the upstream region and three were covered by mCHH DMRs in the downstream region. Majority of these genes (82%, 79/96) were hypomethylated in the root tissue, suggesting this subset of 79 genes may function in stress-related pathways by methylation modification. Additionally, 148 genes were identified that function related to oxidation–reduction processes (highlighted in red and yellow in Additional file 13: Figure S5). These genes were covered by mCHH DMRs in both the flanking and body regions (Fig. 4c and Additional file 13: Figure S5), and all were found to have CHH hyper-methylation in the root (Additional file 14: Data S4).

### Effects of DNA methylation on gene expression

**Fig. 5:**
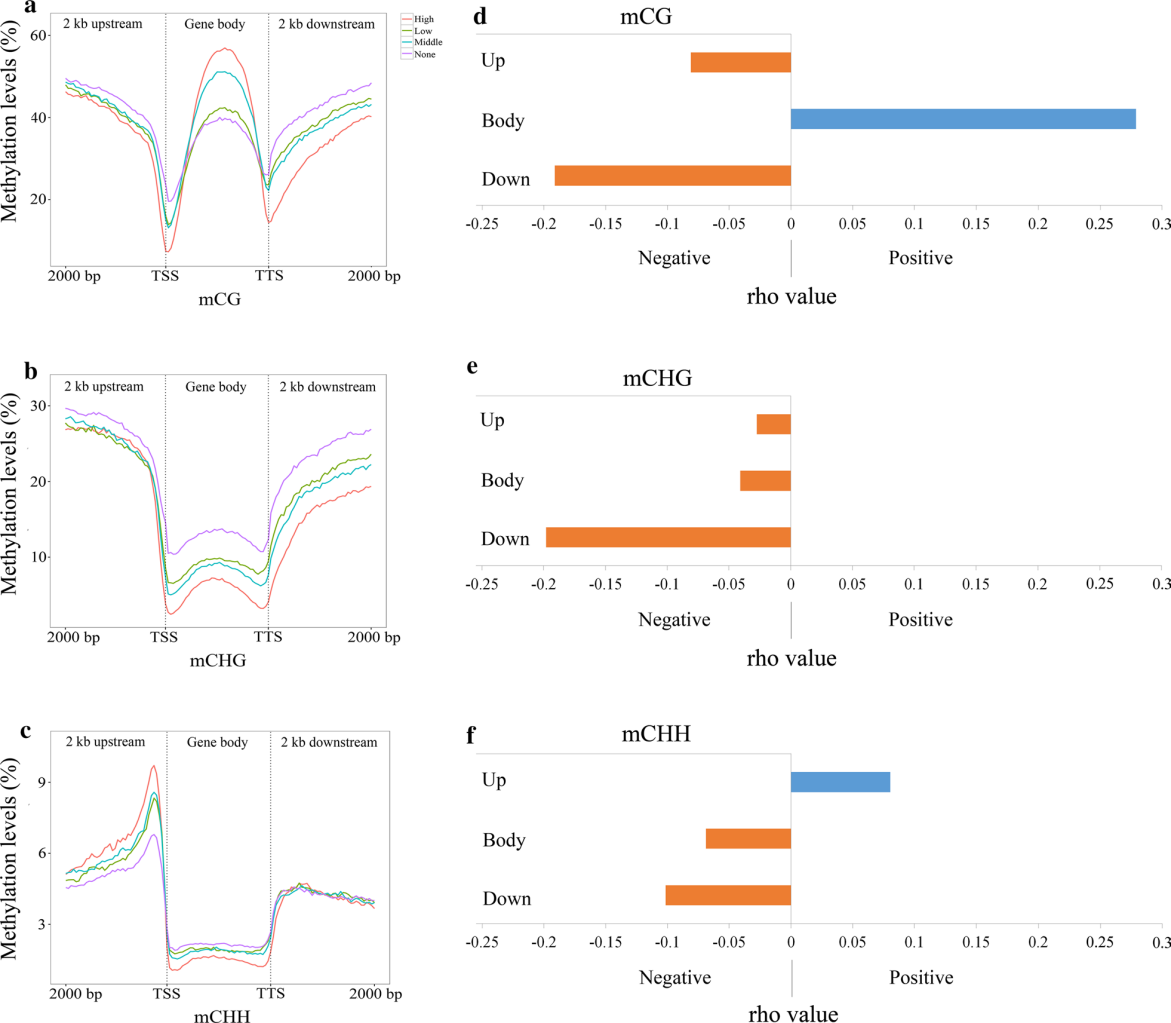
Effect of DNA methylation of mCG (**a**), mCHG (**b**), and mCHH (**c**) on global gene expression in switchgrass. *X*-axis indicates distance from 2-kb upstream to 2-kb downstream, and *y*-axis indicates methylation level (%). TSS and TTS means transcript start site and transcript terminate site, respectively. Red, blue, green, and purple colors mean high-expressed group, middle-expressed group, low-expressed group, and non-expressed group, respectively. **d**–**f** The Spearman analysis between DNA methylation, and gene expression in mCG (**d**), mCHG (**e**), and mCHH (**f**) sites, respectively. *rho* > 0, means positive correlation; *rho* < 0, means negative correlation

To further determine whether DNA methylation influences gene expression in switchgrass, total RNAs were extracted from the same sets of leaf samples and subjected to RNA-sequencing. A total of 150,545 transcripts were assembled from the RNA-seq data and these transcripts were grouped into four categories according to the criteria of Lu et al. [42]. These categories included a non-expressed group (reads per kilobase per million reads mapped [RPKM] ≤ 1), a low-expressed group (1 < RPKM ≤ 10), a middle-expressed group (10 < RPKM ≤ 100), and a high-expressed group (RPKM > 100) (Additional file 15: Table S6 and Fig. 5a–c).

In the 2-kb regions upstream and downstream of genes, the category of non-expressed gene group contained the highest levels of mCG. Alternatively, the methylation levels at this site were the lowest for the high-expressed gene group (Fig. 5a). This indicates a negative association between mCG levels and gene expression in the flanking regions (Fig. 5). In contrast, the mCG levels found to occur in the gene body regions were positively correlated to the gene’s expression level (Fig. 5a). For mCHG, the methylation levels in the gene body and downstream regions were found to be negatively correlated with gene expression. Correspondingly, the non-expressed gene category contained the highest mCHG levels in the upstream, gene body, and downstream regions (Fig. 5b). For the mCHH sites, methylation levels in the upstream region close to transcription starting sites (TSS) were positively correlated with gene expression (Fig. 5c). There was a negative association, however, between gene expression and mCHH levels in the gene body regions, although the changes in methylation levels were minimal across the different categories for this region (Fig. 5c). No obvious relation was observed between mCHH levels in the downstream region (2-kb or less) of genes or in the proximity close to transcription termination sites (TTS) (Fig. 5c).

Spearman correlation analysis was performed to discern statistically the relationships between DNA methylation and gene expression within the 2-kb flanking regions of the protein-coding genes [43] (Fig. 5d–f; Additional file 16: Table S7). The results from that analysis found similar associations between DNA methylation levels and gene expression patterns (Fig. 4a–c). For the mCG sites, there was a significantly different positive correlation of methylation across the gene body region (*rho* = 0.279, *p* = 2.25E −267), whereas mCG in the upstream (*rho* = − 0.080, *p* = 2.42E−51) and downstream (*rho* = − 0.191, *p* = 2.02E−289) regions exhibited a significantly different negative correlation (Fig. 4d; Additional file 16: Table S7). The mCHG sites were found to be negatively and significantly correlated with gene expression in the body and flanking regions; however, the absolute *rho* of the upstream (*rho* = − 0.027, *p* = 5.25E − 07) and body regions (*rho* = − 0.045, *p* = 1.14E − 17) were lower than that of the downstream regions (*rho* = − 0.198, *p* = 2.71E − 311). This suggests a weaker correlation between CHG methylation and gene expression in the upstream and body regions than in the downstream regions (Fig. 5e; Additional file 16: Table S7). For mCHH sites, the upstream region exhibited a positive and significant correlation (*rho* = 0.078, *p* = 5.05E -47), whereas the gene body and downstream regions displayed negative and significant correlations (*rho* = − 0.068, *p* = 7.6E−39 in the body; *rho* = − 0.107, *p* = 6.84E−92 in the downstream) (Fig. 5f). Regression analysis, based on a zero hurdle model, was conducted to verify the associations between DNA methylation and gene expression [44]. Those results found that the mCG sites of the gene body and the mCHH sites of upstream regions were significantly and positively associated with gene expression (model coefficient = 0.01255, *p* = 2.83E−244 for the mCG sites; model coefficient = 0.02431, *p* = 1.63E−41 for the mCHH sites). Other methylated regions, which had zero model coefficients less than 0, had negative associations with gene expression. The predictivity of the regression model was tested using a tenfold cross-validation method [45] and generated small positive *Q*^2^ values, suggesting a limited ability of the model to predict gene expression (Additional file 17: Table S8). This is expected as gene expression is regulated by a number of different genetic and environmental factors interacting with each other.

Collectively, the Spearman and regression analyses determined that the mCG and mCHH sites were positively correlated with gene expression in the gene body and upstream regions, respectively. The other regions analyzed exhibited negative correlations. Despite these results, most regions had very weak correlations according to previously established criteria (www.statstutor.ac.uk/resources/uploaded/spearmans.pdf; the criteria ‘very weak’ was defined as the absolute value of *rho* < 0.2) in the Spearman analysis. Regardlessly, these weak correlations supported all of the relationships predicted through RNA-seq analysis with the exception that no association was found in the downstream region for mCHH sites (Fig. 5a–c).

### mCHH is positively correlated with siRNA expression in genic regions

**Fig. 6:**
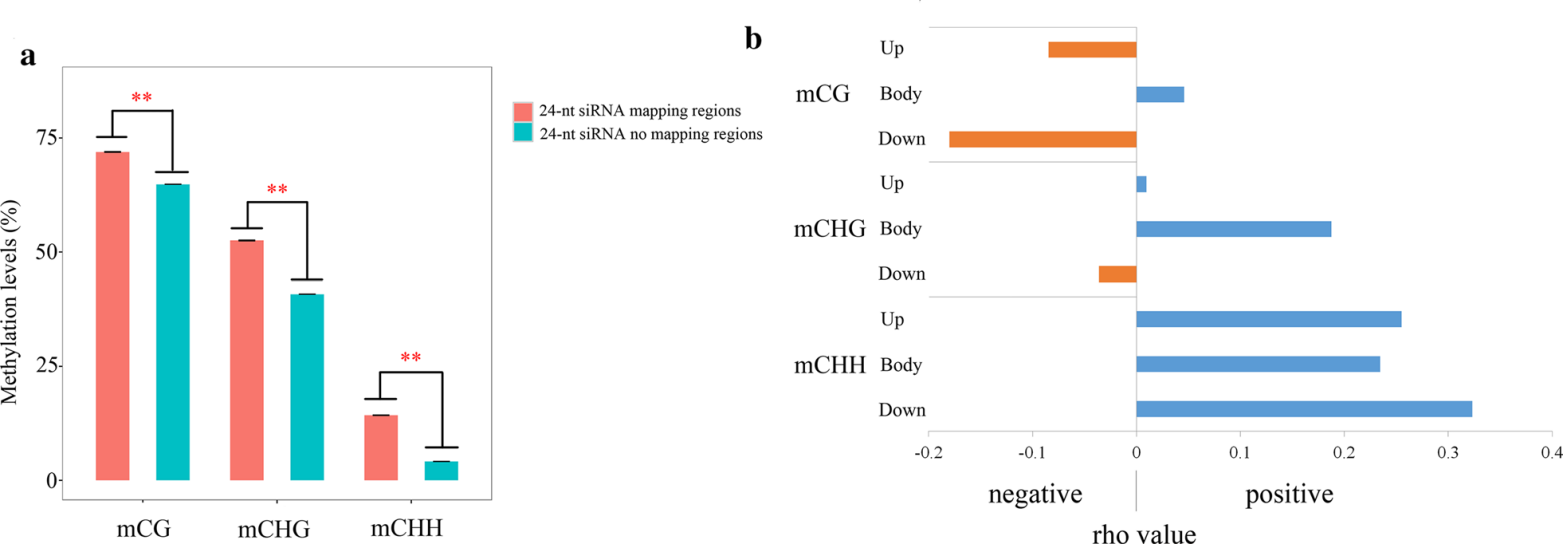
Association between 24-nt siRNA and DNA methylation in switchgrass. **a** Comparison of DNA methylation levels between siRNA uniquely mapping region and without that region in the whole genome-scale. ** represents highly significant difference (*p* < 0.01). **b** The Spearman analysis between DNA methylation, and siRNA expression surrounding genic region. *rho* > 0, means positive correlation; *rho* < 0, means negative correlation. Up, body, and down mean upstream, body, and downstream regions of genes

siRNAs can direct the de novo methylation of cytosine of complementary DNA sequences [46, 47]. In this study, switchgrass miRNAs were profiled using high-throughput sequencing of leaf tissue to elucidate the relationship between DNA methylation and siRNA expression. Switchgrass siRNAs were extracted from the miRNA data. The 24-nt class was the most abundant group (Additional file 18: Figure S6). The 24-nt siRNAs were mapped to the switchgrass reference genome, and the average methylation levels in the regions mapped with or without a siRNA were calculated (Fisher’s exact test, *p* < 0.05; Additional file 19: Table S9). The levels of mCG, mCHG, and mCHH in the siRNA-covered regions were significantly (*p* < 0.01) higher than in the regions not targeted by any siRNAs (Fig. 6a). Therefore, these three methylation sites might positively correlate to siRNAs on a whole-genome scale.

Spearman correlation analysis was employed to further understand if the relationship between siRNA and DNA methylation occurs within the gene body and the 2-kb flanking regions surrounding the genes (Fig. 6b; Additional file 20: Table S10). The mCG sites exhibited a positive and significant correlation in the gene body region (*rho* = 0.044, *p* = 1.68E−07), but negative correlations in the upstream (*rho* = − 0.085, *p* = 2.77E−46) and downstream regions (*rho* = − 0.184, *p* = 3.26E−192). Similarly, the mCHG sites were found to have positive and significant correlations between siRNA and DNA methylation in the gene body region (*rho* = 0.185, *p* = 1.39E−110), and a negative correlation in the downstream flanking region (*rho* = − 0.035, *p* = 2.56E−08). No correlation between the two elements was determined for this site for the upstream gene region (*p* > 0.05). In contrast, the gene body (*rho* = 0.228, *p* = 3.42E−214), as well as the flanking regions (upstream: *rho* = 0.253, *p* = 1.02E−171; downstream: *rho* = 0.317, *p* = 2.02E−115), were found to have positive and significant correlations at the mCHH sites. Regression analysis was conducted based on a negative binomial model [44] to validate these relationships. For mCG sites, regression analysis identified positive and significant correlations in the gene body (model coefficient = 0.00845, *p* = 1.84E−69) and upstream regions (model coefficient = 0.00177, *p* = 6.52E−11), and a negative and significant correlation in the downstream region (model coefficient = − 0.00412, *p* = 1.27E−46). The mCHG sites displayed positive correlations in the upstream and body regions (model coefficient = 0.00558, *p* = 2.07E−60 for the upstream region; model coefficient = 0.00835, *p* = 1.98E−33 for the body region); however, there was no significant correlation in the downstream region (*p* > 0.05). In contrast, mCHH sites in all regions exhibited positive and significant correlations (model coefficient > 0, *p* < 0.05). Through the tenfold cross-validation analysis, the model showed a limited power (small positive *Q*^2^ values) to predict the siRNA expression (Additional file 21: Table S11).

Collectively, the levels of mCHH were positively and significantly correlated with siRNA expression in both the gene body and flanking regions. The mCHH sites had overall higher correlation *rho* than the mCG and mCHG sites in the Spearman analysis (Fig. 6b). Taken together, these results suggest that RdDM plays a critical role in directing formation of mCHH in switchgrass.

### Identification of long non-coding RNAs and their negative relation to DNA methylation

LncRNAs have been shown to be closely associated with DNA methylation [21]. A total of 9244 lncRNAs were identified in the leaf tissue. These lncRNAs ranged in length from 200 base pair (bp) to 3023 bp with an average of 399 bp. The lncRNAs were found to be close to adjacent genes and had an average distance of 12,632 bp away from their flanking genes. This number is higher than that found in similar studies for rice (871 bp) and maize (6761 bp) [48]. The switchgrass lncRNAs could be classified into six groups, the majority of which were classified as the intergenic group (8217; 88.89%). This was followed by the bidirectional (470; 5.08%) and antisense groups (381; 4.12%). The rest of the lncRNAs (< 100) were classified into three additional small groups: intronic sense (1.07%), sense (0.77%), and intronic antisense (0.06%) (Additional file 22: Figure S7). To determine if the switchgrass lncRNAs have homologous genes in rice and maize, the identified 9244 lncRNAs were BLAST against 8594 rice and 4403 maize lncRNAs in CANTATAdb (http://cantata.amu.edu.pl/index.html#about) [49]. Surprisingly, only 63 (0.68%) and 308 (3.33%) switchgrass lncRNAs were found to have homologous genes in rice and maize, respectively. Therefore, most of the lncRNAs identified in this study are unique to switchgrass. The identified lncRNAs were then BLAST against the Rfam family database (http://rfam.xfam.org/) [50]. Seven lncRNAs (Additional file 23: Table S12) were found to belong to the HAR1A [51], Xist_exon1 [52], SOX2OT_exon2 [53], H19_3 [54], ZEB2_AS1_2 [55], and RFPL3 [56] non-coding RNA families, which have been characterized in animal and human systems [57]. The function of these seven lncRNAs in plants, however, still needs to be evaluated. The lncRNAs were aligned to miRbase (v21). Two lncRNAs, TCONS_00103604 and TCONS_00155383, were identified that could potentially be the precursors of miR169 and miR171, respectively (Additional file 24: Table S13). According to the gene annotations of these miRNA targets, three pectinesterase inhibitor genes are targeted by miR169 and an additional two genes are believed to be involved in responses to stressful conditions. The genes targeted by miR171 were primarily predicted to be Scarecrow (SCR) genes (Additional file 24: Table S13).

**Fig. 7:**
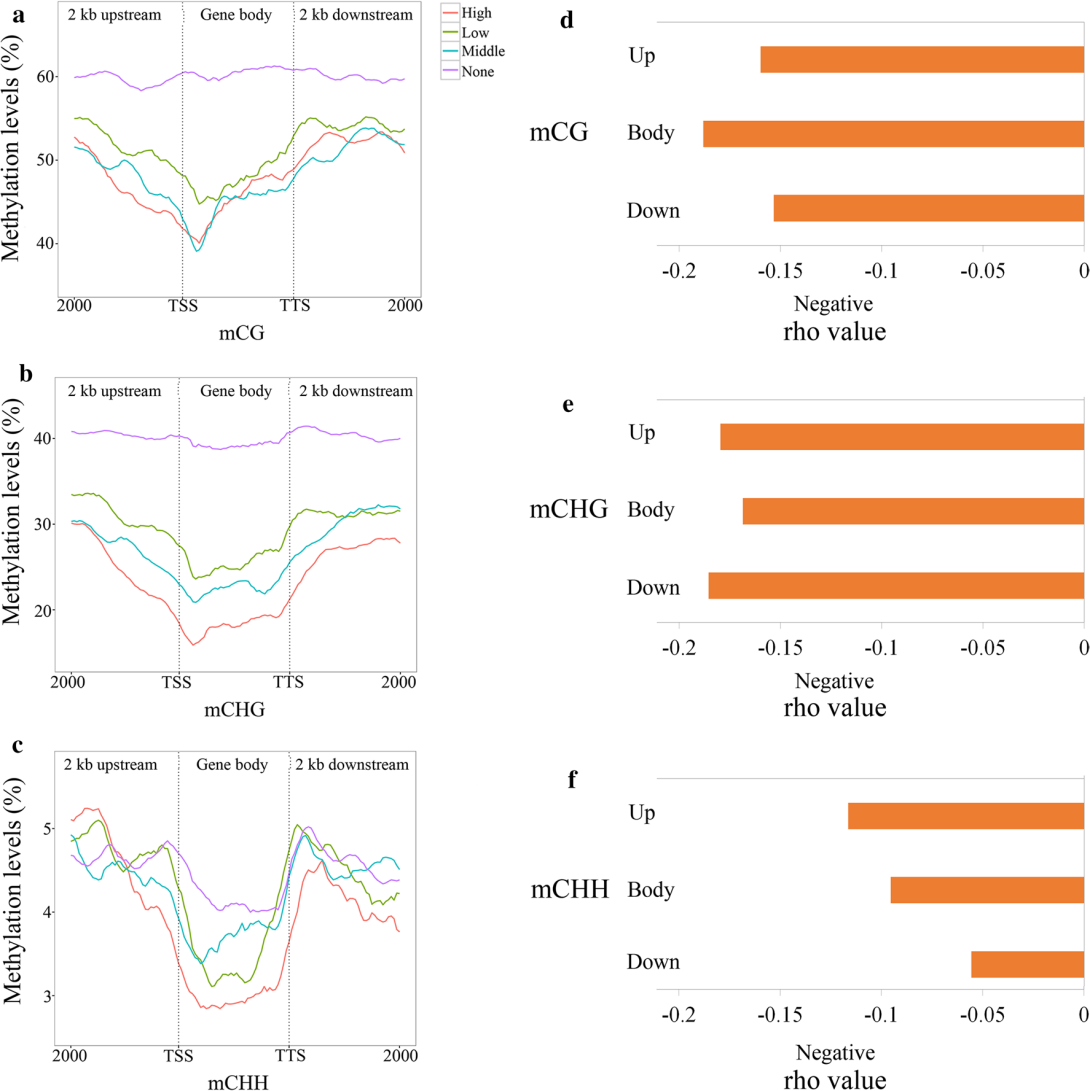
Effect of DNA methylation of mCG (**a**), mCHG (**b**), and mCHH (**c**) on global lncRNA expression in switchgrass. *X*-axis indicates distance from 2-kb upstream to 2-kb downstream, and *y*-axis indicates methylation level (%). TSS and TTS means transcript start site and transcript terminate site, respectively. Red, blue, green, and purple colors mean high-expressed group, middle-expressed group, low-expressed group, and non-expressed group, respectively. **d**–**f** The Spearman analysis between DNA methylation, and lncRNA expression in mCG (**d**), mCHG (**e**), and mCHH (**f**) sites, respectively. *rho* < 0, means negative correlation

DNA methylation allows for an organism to quickly respond to environmental stimuli by altering levels of mRNA expression [58]. LncRNAs have structural and functional regulations similar to mRNAs [59–61]. Thus, the expression of lncRNAs might also be regulated by DNA methylation. The 9244 switchgrass lncRNAs were classified into four groups based on their expression levels (high-expressed group, middle-expressed group, low-expressed group, and non-expressed group) using the same criteria as shown in Fig. 4a–c [42]. DNA methylation association patterns at mCG and mCHH sites were found to be different between mRNA and lncRNA genes. In general, the non-expressed lncRNA group was hyper-methylated on the mCG and mCHG sites, which was not found for the mRNA genes (Figs. 5a, b, 7a, b). In the upstream flanking region, the non-expressed lncRNAs displayed the highest mCG levels. In contrast, the high-expressed lncRNAs were hypomethylated at this site (Fig. 7). These results suggest that mCG in the upstream flanking region might be negatively associated with gene expression of lncRNAs (Fig. 7a). In the gene body and downstream flanking regions, the non-expressed group exhibited the highest methylation levels, and the high-and middle-expressed lncRNAs displayed similar methylation patterns (Fig. 7a). Additionally, the mCHG sites of lncRNAs had comparable patterns with mRNAs, and the mCHG levels in both the flanking and body regions were found to be negatively associated with gene expression (Figs. 5b, 7b). In the gene body region, mCHH levels were determined to be negatively associated with gene expression; however, this was not found to be true in the flanking regions (Fig. 7c).

We also performed Spearman and regression analysis (based on a zero hurdle model) to verify the predicted relationships of lncRNAs within the 2-kb flanking regions (Fig. 7d–f; Additional file 25: Table S14; Additional file 26: Table S15). For all three methylation contexts, these two different statistical approaches both showed that methylation levels had significant negative correlations to lncRNA expression in the 2-kb flanking regions as well as the gene body region, except for the mCHH sites of downstream region (*p* > 0.05) in the regression analysis. This predictivity of regression model was also tested via the tenfold cross-validation method. The yielded small positive *Q*^2^ values from the model exhibited a restrictive ability to predict the lncRNA expression. (Additional file 25: Table S14; Additional file 26: Table S15). Overall, these results supported the findings of a negative relationship between lncRNA expression and mCHG in the flanking and body regions, mCG in upstream region, and mCHH in the gene body region (Fig. 7a–c).

## Discussion

Switchgrass is a largely self-incompatible species [62, 63]. The relatively complex genome of switchgrass has hampered the development of whole-genome sequencing on this important biofuel crop. Recently, the latest version of the switchgrass genome (v4.1) was released, which contains high-chromosome-scale contiguity that can allow for researchers to apply modern omics techniques to this plant species. A recent report generated a profile of DNA methylation on genomic features of two switchgrass genotypes, AP13 (Alamo clone) and VS16 [64]. In general, this study found similar methylation patterns for switchgrass as the ones identified in the current study: methylation levels of genic flanking regions were higher than in the gene body region and TEs themselves were more highly methylated than the upstream and downstream regions flanking them. However, this previous study did not examine the association between the DNA methylome and the transcriptome of switchgrass. To resolve relationships between these two omics, we conducted genome-wide single-base resolution methylome, lncRNA, miRNA, and mRNA sequencing and aligned the data to the latest switchgrass genome (v4.1) with a relatively high assembly quality.

### Switchgrass has unique DNA methylation features but also shares general DNA methylation profiles with other plant species

In this study, the methylation distribution across the gene body was analyzed. It was discovered that the methylation levels of intron regions were higher than other regions in the switchgrass genome (Fig. 1c). These results are not consistent with those found in C_3_ model plants, such as *Arabidopsis*, rice, and poplar [65]. In addition, the mCG sites were the most dominant in switchgrass, followed by mCHG and mCHH. The levels of mCG and mCHG were the highest in the gene body region (Fig. 2a), which is consistent with observations in most plants [34, 36, 42, 66, 67]. The gene flanking regions in switchgrass, however, displayed higher mCG levels than in the gene body region (Fig. 2a, c). This is a stark contrast to methylation patterns in most other plant species [32, 68, 69] and suggests that more methylation modifications, and subsequently more epigenetic mechanisms, may occur in the gene flanking regions of switchgrass. The average methylation level of TEs was higher than that of the genic regions in switchgrass, which is consistent with results found in most other plant species [17, 20, 32]. Additionally, we discovered that the terminal chromosome regions were less methylated at the mCG and mCHG sites, which is similar to rice [17, 20, 32]. It has been reported that the largest number of repeats with high methylation levels exists in telomeric heterochromatin regions [70]. Therefore, the lower methylation levels detected in the terminal chromosome regions in this study may indicate that these terminal regions do not cover telomeric heterochromatin, which could be caused by sequence limitations of the current switchgrass genome. Overall, despite having a polyploid genome and several methylation differences, switchgrass does indeed share some DNA methylation profiles with the majority of plant species.

### Identification of stress-related genes covered by DMRs

Switchgrass has a relatively large and strong root system that allows it to be more tolerant to various abiotic stresses [71]. In addition, identification of DMRs between roots and leaves might help find stress-related genes controlled by DNA methylation.

A total of 96 genes covered by DMRs were found to be involved in stimulus-related GO processes. For these genes, 82% were mCG hypomethylated in the downstream region and mCHG hypomethylated in the upstream region in the root tissues (Additional file 12: Data S3). Since the levels of mCG in the downstream regions and mCHG in the upstream regions were negatively correlated with gene expression, these 79 genes may possibly be upregulated by hypomethylation in the root tissue (Fig. 4c). Additionally, nine of the 79 hypomethylated genes were annotated to be auxin response factors and Small Auxin Upregulated RNA (SAUR) genes, which have been shown to be involved in stress defense responses [72]. These data suggest that switchgrass roots can quickly respond to environmental stresses, as well as maintain proper growth and development, by changing methylation on some auxin and stress-responsive genes. Some of these 79 genes also contained specific functional domains. For example, two genes contained Mildew resistance Locus O (MLO) domains, which are known to function as negative regulators of broad spectrum disease resistance [73]. In addition, one gene had a flavin adenine dinucleotide (FAD)-binding domain that is related to DNA photolyase [74]. Finally, one gene possessed a DOS2-like protein (BSD) domain, which has been reported in a rice *OsBSD* gene and has been shown to have a crucial role in plant growth [75] (Additional file 12: Data S3).

The mCHH DMRs covered a different subset of 148 switchgrass genes, which were identified and annotated to function in oxidation–reduction processes. All of these genes were hyper-methylated in the root tissues (Additional file 14: Data S4). The reactive oxygen intermediates (ROIs) produced as signaling molecules by these gene products, along with each gene’s role in oxidation–reduction reactions, could control switchgrass response to various processes including pathogen defense, programmed cell death, abiotic stress response, and systemic signaling [76]. Unfortunately, the mRNA expression profiles of these genes were not examined in the switchgrass root tissue. Therefore, additional studies are needed to determine if all or most of these 148 DMR genes have higher or lower expression levels in roots compared to other plant tissues. It would be interesting to further characterize if these genes do indeed play roles in switchgrass tolerance to abiotic stress.

### DNA methylation can either positively or negatively regulate gene expression depending on the methylation sites in different genic regions

DNA methylation is not simply an inhibitor of gene expression [17, 26, 27, 29, 30]. In the gene promoter regions, the presence of DNA methylation usually suppresses transcription initiation, although low methylation levels occurring in these regions may promote gene expression. Our results found a negative relationship between DNA methylation of CG and CHG sites in the gene promoter regions and gene expression, suggesting that methylation of promoters represses gene expression in switchgrass (Fig. 5a, b). This observation was consistent with studies in *Arabidopsis* [26, 30, 67] and rice [26, 30, 67]. These results support previous studies that found that promoter symmetrical methylation is a common epigenetic mechanism controlling gene expression in eukaryotes. Interestingly, the mCHH levels in the upstream gene regions were positively correlated with gene expression (Fig. 5c). This could be attributed to TEs located close to or within the promoters of nearby genes [77, 78]. It is possible that RNA polymerase II- or IV-mediated transcription, which is initiated by TEs, can spread to the nearby gene regions and subsequently increase expression of the nearby genes [46, 79]. We observed that a higher number of 24-nt siRNAs target to TEs that are closer to genes than those TEs that are located at the intergenic regions (Additional file 27: Figure S8). Studies have shown that siRNAs can induce mCHH methylation through the RdDM pathway [80]. The abundance of siRNAs in these areas can result in a higher level of mCHH. This would explain the positive association between mCHH sites and gene expression in the upstream promoter region (Fig. 5c).

In the gene body region, the mCG sites were positively correlated with gene expression (Fig. 5a). This phenomenon could be attributed either to DNA methylation directly/indirectly preventing the initiation of intragenic promoters or to DNA methylation interfering with the activities of repetitive DNAs within the transcriptional unit [81]. In contrast, a negative association was found to exist between gene expression and methylation levels of mCHG and mCHH in the gene body regions, suggesting that the mCHG and mCHH in the actively transcribed genes may not inhibit gene transcription (Fig. 5b, c) [82, 83].

The data from this study also revealed that mCG and mCHG sites in the transcriptional termination regions (TTR) were negatively correlated with gene expression (Fig. 5a, b). The differences in methylation levels among four categories (high, low, middle, and non-expressed genes) near the TTR regions were substantially greater than in the promoter regions. These results imply that mCG and mCHG in the TTR region might have a more significant role in the regulation of gene expression than their corresponding sites in the promoter region (Fig. 5a, b).

Overall, the mCG and mCHH were found to be positively related to gene expression in the gene body and upstream flanking regions, respectively. These results differed from other regions that displayed a negative correlation (Fig. 5). Taken together, these findings suggest that DNA methylation can either positively or negatively regulate gene expression and control of these processes depends on the methylation sites in different genic regions.

### The 24-nt siRNAs induce increased mCHH levels in the genic regions of switchgrass

RdDM is a process by which 24-nt siRNAs direct the de novo methylation of cytosine of complementary DNA sequences. The de novo methylation of DNA at all mCG, mCHG, and mCHH contexts is usually performed by domains rearranged methylase 2 (DRM2) through the RdDM pathway [84]. In this study, mCHH levels, but not mCG and mCHG levels, were found to have a positive correlation with siRNA expression (Fig. 6b). After the initial methylation of DNA, mCG and mCHG sites could be sustained by copying the information from the parental strand after DNA replication. In comparison, most mCHH sites need to be maintained de novo after each round of DNA replication through the RdDM pathway [80]. Therefore, only the production of mCHH sites was positively correlated with siRNA expression in the switchgrass genic region, suggesting that 24-nt siRNAs can significantly increase mCHH levels in the canonical RdDM pathway. However, on a whole-genome scale, these three methylation contexts might all be positively correlated with siRNA expression (Fig. 6a), suggesting that siRNAs might activate mCG and mCHG in the intergenic and TE regions rather than the genic regions.

### Identification of two functional precursory lncRNAs of miRNAs

Previous studies suggest that lncRNAs could be precursors of miRNAs [85, 86]. By aligning the lncRNAs identified in this study to miRbase (v 21), two lncRNAs TCONS_00103604 and TCONS_00155383, were found that could be the precursors of miR169 and miR171, respectively (Additional file 24: Table S13). Based on the annotations of the genes targeted by these miRNAs, we found three genes targeted by miR169 identified as pectinesterase inhibitors (Additional file 24: Table S13). Pectinesterase can convert components of the plant cell wall to pectic acid and may have a role in the cellulose biosynthetic pathway [87, 88]. Correspondingly, the chemical and structural features of switchgrass cell walls can have a significant effect on biofuel yields [89, 90]. Therefore, the regulation of miR169 and its precursor sequence may contribute to biofuel yields in switchgrass. miR169 might also target two genes that code for stress-responsive proteins, indicating that miR169 and its precursor might also regulate stress tolerance in switchgrass. The targets of miR171 were primarily predicted to be the SCR genes (Additional file 24: Table S13), which are expressed in hypocotyl, inflorescence, and stem tip tissues [91]. Recent studies have revealed that mutations in SCR genes can cause proliferation of bundle sheath cells and abnormal differentiation of bundle sheath chloroplasts in maize [92]. In addition, suppression of *LaSCR1* was shown to decrease root numbers in transformed roots of white lupin (*Lupinus albus*) and *Medicago truncatula* [93]. Therefore, miR171 and its precursor (TCONS_00155383) are likely to be involved in stem and root development in switchgrass.

## Conclusion

This study utilized a combination of omics techniques to conduct genome-wide single-base-resolution methylome, lncRNA, miRNA, and mRNA sequencing in switchgrass. The results presented here support that siRNAs positively regulate DNA methylation at mCHH sites and that DNA methylation may interfere with gene and lncRNA expression in the polyploid switchgrass genome. In addition, a total of 96 genes were identified that are covered by DMRs in leaf and root tissues. These genes are believed to be involved in stimulus-related GO processes and 79 of them were hypomethylated in the root tissue. We also identified 9244 novel lncRNAs in switchgrass and predicted two lncRNA precursors of miRNAs that may function in cellulose biosynthesis, stress regulation, and stem and root development. Overall, the successful DNA methylome and transcriptome sequencing of switchgrass presented in this study provides a reference for other highly heterozygous Poaceae grasses with similar characteristics. These results could also serve as genomic resources for identifying further methylation patterns and additional non-coding RNAs analysis in switchgrass.

## Experimental procedures

### Plant materials

The switchgrass cv. Alamo was propagated through tiller-splitting and planted in pots (0.25 m diameter × 0.4 m tall) containing 1500 g soil (pH 5.37, 1.26% organic qualitative content, 98.38 mg/kg N, 4.48 mg/kg P, and 328.22 mg/kg K). The plants were maintained in a greenhouse (Wenjiang, Sichuan, China) at 28 °C/20 °C (day/night) with a photoperiod of 16 h/8 h (day/night). Four months after transplanting and approximately 1 week before the emergence of flower primordium (E5 stage), the flag leaves and roots of six individual plants were collected [94]. The leaf and root tissues were frozen in liquid nitrogen and stored at − 80 °C before total DNAs and RNAs were extracted.

### Methylation data analysis

#### DNA extraction

The flag leaves and roots were pooled from six random individuals with equal masses for each one and were ground to a fine powder in liquid nitrogen. Genomic DNA was extracted using a plant genomic DNA kit (Tiangen, China) following the manufacturer’s instructions. The DNA integrity and concentration were measured by agarose gel electrophoresis and NanoDrop spectrophotometer, respectively.

#### Library construction and sequencing

Bisulfite sequencing libraries were prepared using the TruSeq Nano DNA LT kit (Illumina, San Diego, CA, USA) as described in Du’s study [95]. The genomic DNAs were then fragmented into 100–300 bp by sonication (Covaris, USA) and purified using a MiniElute PCR Purification Kit (QIAGEN, Silicon Valley Redwood City, CA, USA). The fragmented DNAs were end repaired and a single ‘A’ nucleotide was appended to the 3′ end of each fragment. After ligating the DNAs to the sequencing adapters, the genomic fragments were bisulfite converted via a Methylation-Gold kit (ZYMO, Murphy Ave. Irvine, CA, USA). The converted DNA fragments were PCR amplified and sequenced as paired-end reads using the Illumina HiSeq™ 4000 platform by the Gene Denovo Biotechnology Co. (Guangzhou, China).

#### Data filtering

The raw reads generated from the Illumina HiSeq™ 4000 were filtered to get high-quality reads using fastq-mcf (v1.04) tool [96] according to the following principles: (1) reads with more than 10% of unknown nucleotides (N) were removed, and (2) reads with more than 40% of low-quality (*Q*-value ≤ 20) bases were removed.

#### Methylation level analysis

We mapped the clean reads to the switchgrass reference genome (https://phytozome.jgi.doe.gov/pz/portal.html#!info?alias=Org_Pvirgatum_er;V4.1) using BSMAP software (v 2.90) [97] with the default parameters. The effective coverage of cytosine was defined as the number of coverage reads on cytosine ≥ 1. The effective coverage rate of cytosine was calculated based on the ratio of the number of effective coverage of cytosine per all cytosines in one specific region. The mC was called from these effective coverage cytosines, and the methylation level was calculated using a custom script as part of the BSMAP package based on the following ratio: (mC)/(mC + non-mC). This was calculated for the whole switchgrass genome, every chromosome, and for all genomic regions for each of the three methylation contexts (CG, CHG, and CHH). The methylation patterns for the gene body, transposable elements, and flanking upstream and downstream 2-kb regions were plotted using R project software (http://miyoviqo.tha.im/) according to the average methylation levels for each 100-bp interval. ANOVA analysis was applied to test the significance of the average methylation levels of both genic and TE regions between leaf and root tissues [98].

#### Differentially methylated regions analysis

Differentially methylated regions (DMRs) between leaf and root tissues of switchgrass for CG, CHG, and CHH were identified based on the following criteria:

(1) The length of each DMR region was between 40 bp and 10 kb; (2) the distance between adjacent methylated sites was < 200-bp; (3) more than ten reads needed to be present for each cytosine, and more than four reads were need for coverage for each methylated cytosine; (4) more than five methylated cytosines must be present in at least one sample; (5) the fold change of the average methylation level was > 2; (6) Pearson’s chi-square test (*χ*2) value was *p* ≤ 0.05.

#### Enrichment analysis of DMR-related genes

We sorted the putative DMRs covering the gene body regions, the 2-kb flanking regions of genes, and the TEs and conducted GO enrichment analysis via topGO package in R [99] for the DMR-related genes using a hypergeometric test with a corrected *p* ≤ 0.05.

### RNA-seq and data analysis

The six individual plants were pooled into two independent biological replicates, and each replicate contained three individuals. Total RNAs were isolated from the flag leaves of the two replicates using TRIzol reagent (Invitrogen, Carlsbad, NM, USA) according to the manufacturer’s instructions. rRNAs were removed from the samples, retaining only mRNAs and non-coding RNAs. The sequencing libraries were built following Illumina’s standard protocol for RNA-seq library construction as previously described [20], and the libraries were sequenced on an Illumina HiSeq™ 4000 RNA-sequencer at Gene Denovo Biotechnology Co. (Guangzhou, China). To obtain quality reads, the raw data were filtered by removing sequences containing adaptors, low-quality reads (*Q*-value ≤ 20), and reads containing more than 10% of unknown nucleotides (N) using fastq-mcf (v1.04) tool [96]. Bowtie2 [100] was used to map the reads against the ribosome RNA (rRNA) database to remove rRNA mapped reads. The remaining reads were used for further transcriptome analysis and were mapped to the switchgrass reference genome (https://phytozome.jgi.doe.gov/pz/portal.html#!info?alias=Org_Pvirgatum_er; V4.1) using TopHat2 (v 2.0.3.12) with default parameters [101]. Reconstruction of the transcripts was conducted using both Cufflinks [102] and TopHat2. The reconstructed transcripts were re-aligned to the switchgrass reference genome and clustered into 12 categories using Cuffcompare [102]. The abundance of each transcript was quantified using RSEM (v 1.2.19) [103] and transcript expression levels were normalized using RPKM (Reads per kb per Million reads).

### LncRNA data analysis

#### LncRNA prediction

Two programs: CNCI (v2) [104] and CPC [105] (http://cpc.cbi.pku.edu.cn/) were used to evaluate the protein-coding potential of new transcripts with default parameters. Transcripts that had both CPC and CNCI scores less than 0 were chosen as long non-coding RNAs.

#### LncRNA family analysis

Rfam divides non-coding RNAs into families based on their evolution from a common ancestor. Producing multiple sequence alignments of these families can provide insight into their structure and function, similar to the case of protein families. To better annotate lncRNAs at the evolution level, the software Infernal [106] (http://eddylab.org/infernal/) was used in the sequence alignment and lncRNAs were classified by sequence consensus and secondary structures.

#### miRNA precursor prediction

To find potential miRNA precursors, lncRNAs were aligned to miRBase (v 21) using BLAST (v 2.2.25), and those with greater than 90% identity were selected. Additionally, the software miRPara (v 6.2) [107] was employed to predict miRNA precursors using default parameters.

#### Functional group analysis

GO analysis and KEGG analysis were applied to determine the biological roles of the targets genes of identified lncRNAs in switchgrass. GO terms were assigned based on the Gene Ontology (GO) database [108] using GOseq R package [109] and the latest KEGG (Kyoto Encyclopedia of Genes and Genomes) database (http://www.genome.jp/kegg/) using KOBAS [110]. The *p* value (Hypergeometric-P value) cut-off was set to 0.05.

### Small RNA-seq data analysis

RNAs extracted from the flag leaves of the two biological replicates were used for small RNA-sequencing. Small RNA molecules with a size range of 18–30 nt were enriched by polyacrylamide gel electrophoresis (PAGE) and libraries were built using the NEBNext multiplex small RNA library prep set (NEB#E7300, Ipswich, MA, England) based on the manufacturer’s protocol. The small RNAs were then sequenced using an Illumina HiSeq 2500 by Gene Denovo Biotechnology Co. (Guangzhou, China). Raw reads were filtered by removing the following: reads without 3′ adapters, reads containing 5′ adapters, reads containing 3′ and 5′ adapters but no small RNA fragment between them, reads containing poly A sequences in the small RNA fragment, and reads shorter than 18-nt (not including adapters). The high-quality sequences were then BLAST (v 2.2.25) [111] against the small RNAs in the GenBank database (Release 209.0) and Rfam database (v 11.0) to remove rRNAs, snRNAs, snoRNAs, scRNAs, and tRNAs. High-quality, clean small RNA sequences were then aligned with the switchgrass reference genome using Bowtie (v 1.1.2) [112] to remove those that mapped to exons or introns. The known switchgrass miRNAs were identified by searching the clean sequences against the miRBase database (Release 21). In addition, the 24-nt reads that did not match any miRNAs were retained and used as siRNAs for subsequent analyses. The siRNA sequences were normalized by calculating the siRNA reads per million based on the total abundance of genome-matched small RNA reads.

### Statistics analysis

The relationships between methylation and mRNAs, siRNAs, and lncRNAs were all analyzed using Spearman and regression analysis. For Spearman analysis, the evaluation of correlation was based on the following criteria (www.statstutor.ac.uk/resources/uploaded/spearmans.pdf): (1) *rho* > 0 equated to a positive correlation, (2) *rho* < 0 signified a negative correlation, (3) very weak was indicated by the absolute value of *rho* < 0.2, (4) weak was indicated by 0.2 ≤ the absolute value of *rho* < 0.4, (5) moderate was determined by 0.4 ≤ the absolute value of *rho* < 0.6, (6) strong was determined by 0.6 ≤ the absolute value of *rho* < 0.79, and (7) very strong was indicated by 0.8 ≤ the absolute value of *rho* < 1.0. The relationships of mRNA, siRNA and lncRNA with methylation were evaluated by modeling gene expression as a function of the number of methylated cytosines in the body of the gene and both flanking regions [44]. For mRNA and lncRNA we used hurdle regression analysis. These two-part models handle read counts with excessive amounts of zeroes (i.e. unexpressed genes) by specifying process for zeroes. This process was modeled with the logistic distribution. The relationship between siRNA and methylation was modeled solely with the negative binomial distribution because zeros were not overrepresented in this case. The prediction power of all regression models was then tested using a tenfold cross-validation method [45]. The input data were randomly and equally divided into ten subsets (tenfold) for the validation. The cross-validate *R*^2^ (also known as *Q*^2^) values were calculated by a formula: *Q*^2^ = 1 − PRESS (predictive error sum of squares)/TSS (total sum of squares).

## Additional files

**Additional file 1: Table S1.** Information on bisulfite sequencing data in switchgrass.

**Additional file 2: Data S1.** Effective coverage rate of cytosine in the chromosome, gene, and repeat regions in both leaf and root tissues of switchgrass.

**Additional file 3: Figure S1.** Sequencing depth and saturation analysis of in leaf (a) and root (b) tissues of switchgrass. The *x*-axis represents the sequencing depth. The y axis represents the percentage for cytosine with a sequencing depth over a specific depth of the whole genome cytosine.

**Additional file 4: Figure S2** Chromosome distribution for root (a) and leaf (b). Methylation level in 80-kb windows throughout chromosomes in the leaf tissue of switchgrass. The red line means ‘−’ strand, and the blue line means ‘+’ strand.

**Additional file 5: Table S2.** The levels of mCG, mCHG, and mCHH and genome size in genomes of different species.

**Additional file 6: Figure S3.** Correlation between genome sizes and methylation levels for different species as *Arabidopsis*, *B. rapa*, *G. max*, *M. truncatula*, *P. trichocarpa*, *Z. mays*, *O. sativa*, *B. distachyum*, and *P. virgatum* (leaf tissue) used in Figure 1e.

**Additional file 7: Figure S4.** Methylation levels of Class I and Class II TEs in the leaf (a) and root (b) of switchgrass.

**Additional file 8: Table S3.** Comparison of methylation levels between class I and class II transposons in leaf and root tissues.

**Additional file 9: Table S4.** Comparison of methylation levels in genic and TE regions between switchgrass leaf and root tissues.

**Additional file 10: Table S5.** Comparison of methylation levels in different TE types between switchgrass leaf and root tissues.

**Additional file 11: Data S2.** GO annotation of genes covered by the DMRs between switchgrass leaf and root tissues.

**Additional file 12: Data S3 .** The methylation levels of 96 genes covered by DMRs and involved in stimulus related GO processes in both leaf and root tissues of switchgrass.

**Additional file 13: Figure S5.** Molecular function for genes covered by the CHH DMRs in the GO annotation in gene upstream (a), body (b), and downstream (c). The colored boxes mean the adjust *p* < 0.05, and the more deeper color, the less adjust *p* values for the boxes.

**Additional file 14: Data S4.** The CHH methylation of DMR covered genes annotated in the oxidation-reduction process and oxidoreductase in both leaf and root tissues.

**Additional file 15: Table S6.** The number of mRNA and lncRNA in the four expression levels.

**Additional file 16: Table S7.** Correlation analysis between methylation levels and gene expression in different genic regions.

**Additional file 17: Table S8.** Regression analysis and 10-fold cross-validation between DNA methylation and gene expression based on zero hurdle model.

**Additional file 18: Table S8.** Length distribution of siRNAs (from 20 to 25 nucleotides) in switchgrass for two biological repeats including Alamo_1 (a) and Alamo_2 (b).

**Additional file 19: Table S9.** Comparison of methylation levels between siRNA uniquely mapping region and without that region.

**Additional file 20: Table S10.** The correlation analysis between gene methylation and siRNA expression.

**Additional file 21: Table S11.** Regression analysis and 10-fold cross-validation between DNA methylation and siRNA expression based on negative binomial model.

**Additional file 22: Figure S7.** Proportion of six different types of lncRNA in switchgrass.

**Additional file 23: Table S12.** Prediction of lncRNA families in switchgrass.

**Additional file 24: Table S13.** Annotation of targets of miR169 and miR171.

**Additional file 25: Table S14**. The correlation analysis between methylation levels and lncRNA expression.

**Additional file 26: Table S15**. Regression analysis and 10-fold cross-validation between DNA methylation and lncRNA expression based on zero hurdle model.

**Additional file 27: Figure S8.** siRNA levels (per bp per million reads) in TEs relative to the distance from the nearest gene.

## Abbreviations

TEs: transposable elements
MiRNAs: microRNAs
lncRNAs: long non-coding RNAs
RdDM: RNA-dependent DNA methylation
DMRs: differentially methylated regions
mCs: methylcytosines
TSS: transcription starting sites
ROIs: reactive oxygen intermediates
DRM2: domains rearranged methylase 2
SAUR: Auxin upregulated RNA
MLO: mildew resistance locus O (MLO)
FAD: flavin adenine dinucleotide
BSD: DOS2-like protein
