# Supplementary material for: siRNAs regulate DNA methylation and interfere with gene and lncRNA expression in the heterozygous polyploid switchgrass
Source: Biotechnol Biofuels. 2018 Jul 24;11:208. doi: 10.1186/s13068-018-1202-0 (PMC6058383; doi:10.1186/s13068-018-1202-0)
Supplement: Supplementary file 1 — Additional file 1: Table S1. Information on bisulfite sequencing data in switchgrass. [file 13068_2018_1202_MOESM1_ESM.docx]

**Table S1** Information on bisulfite sequencing data in switchgrass.

|  | Total reads | Total nucleotides | Q20 percentage | Mapped ratio | Coverage |
| --- | --- | --- | --- | --- | --- |
| Leaf | 411,241,804 | 61,615,469,555 | 97.01% | 81.27% | 29.67X |
| Root | 391,944,518 | 58,756,230,738 | 97.56% | 77.46 % | 26.95X |
