# Supplementary figures and images for: siRNAs regulate DNA methylation and interfere with gene and lncRNA expression in the heterozygous polyploid switchgrass
Source: Biotechnol Biofuels. 2018 Jul 24;11:208. doi: 10.1186/s13068-018-1202-0 (PMC6058383; doi:10.1186/s13068-018-1202-0)

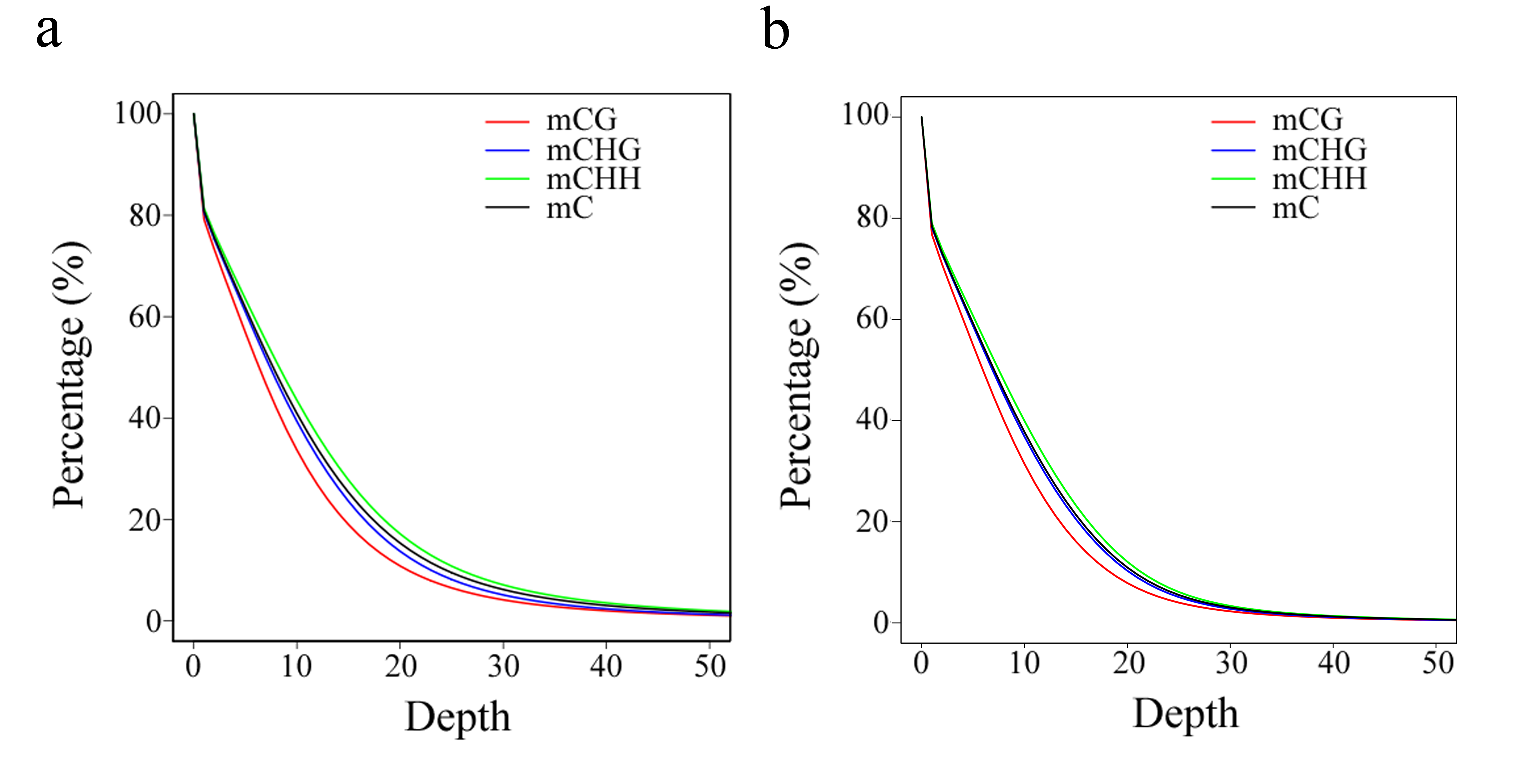

Supplement: Supplementary file 3 — Additional file 3: Figure S1. Sequencing depth and saturation analysis of in leaf (a) and root (b) tissues of switchgrass. The x-axis represents the sequencing depth. The y axis represents the percentage for cytosine with a sequencing depth over a specific depth of the whole genome cytosine. [file 13068_2018_1202_MOESM3_ESM.tif]

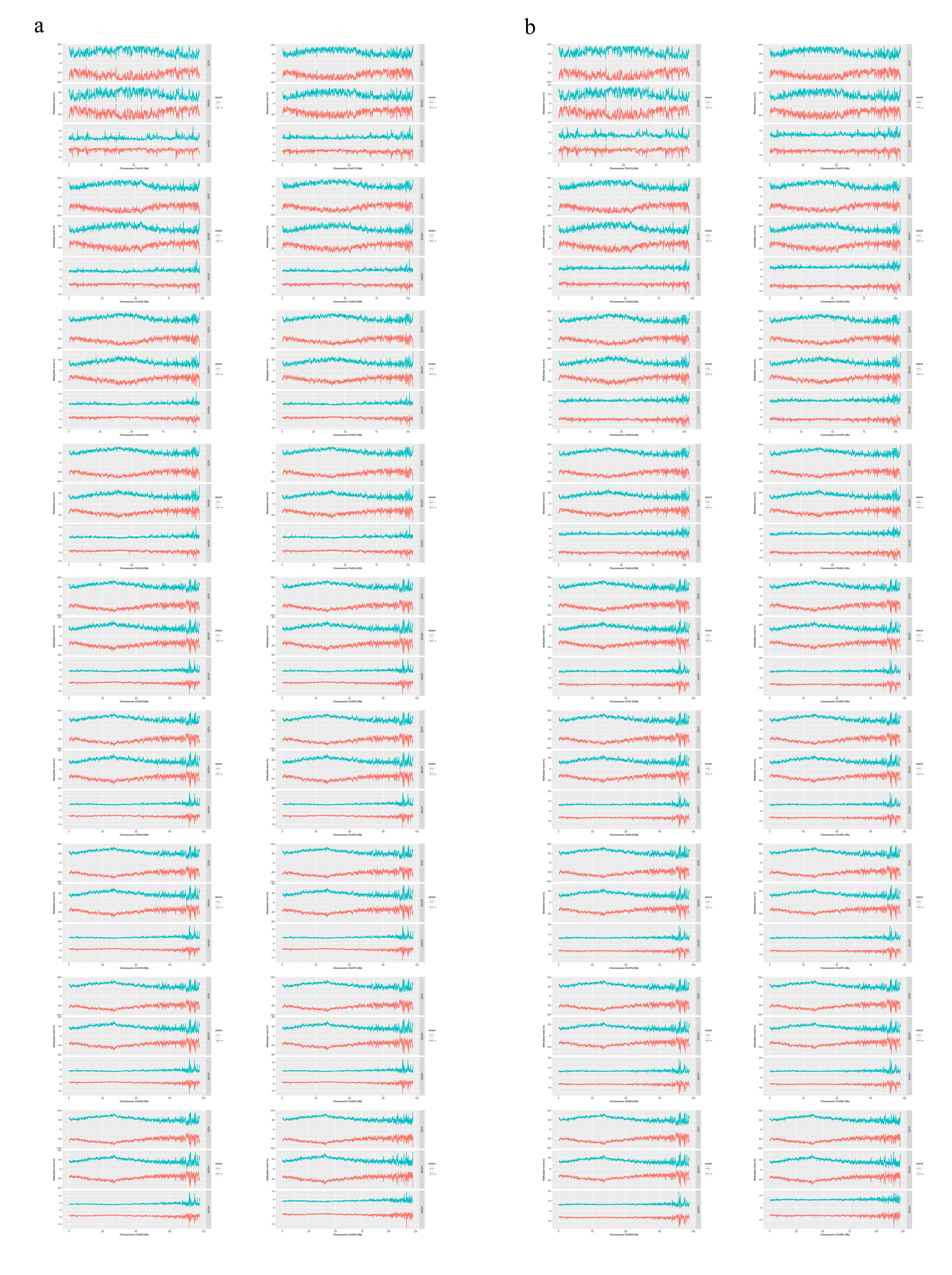

Supplement: Supplementary file 4 — Additional file 4: Figure S2 Chromosome distribution for root (a) and leaf (b). Methylation level in 80-kb windows throughout chromosomes in the leaf tissue of switchgrass. The red line means ‘−’ strand, and the blue line means ‘+’ strand. [file 13068_2018_1202_MOESM4_ESM.tif]

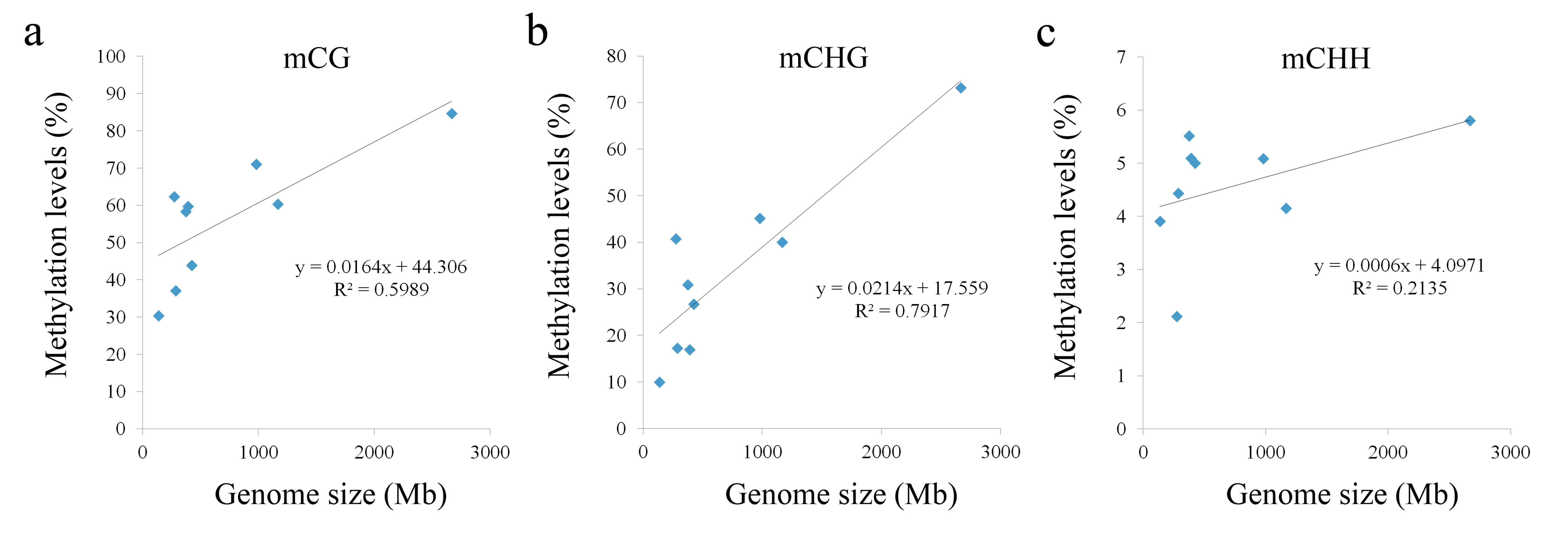

Supplement: Supplementary file 6 — Additional file 6: Figure S3. Correlation between genome sizes and methylation levels for different species as Arabidopsis, B. rapa, G. max, M. truncatula, P. trichocarpa, Z. mays, O. sativa, B. distachyum, and P. virgatum (leaf tissue) used in Figure 1e. [file 13068_2018_1202_MOESM6_ESM.tif]

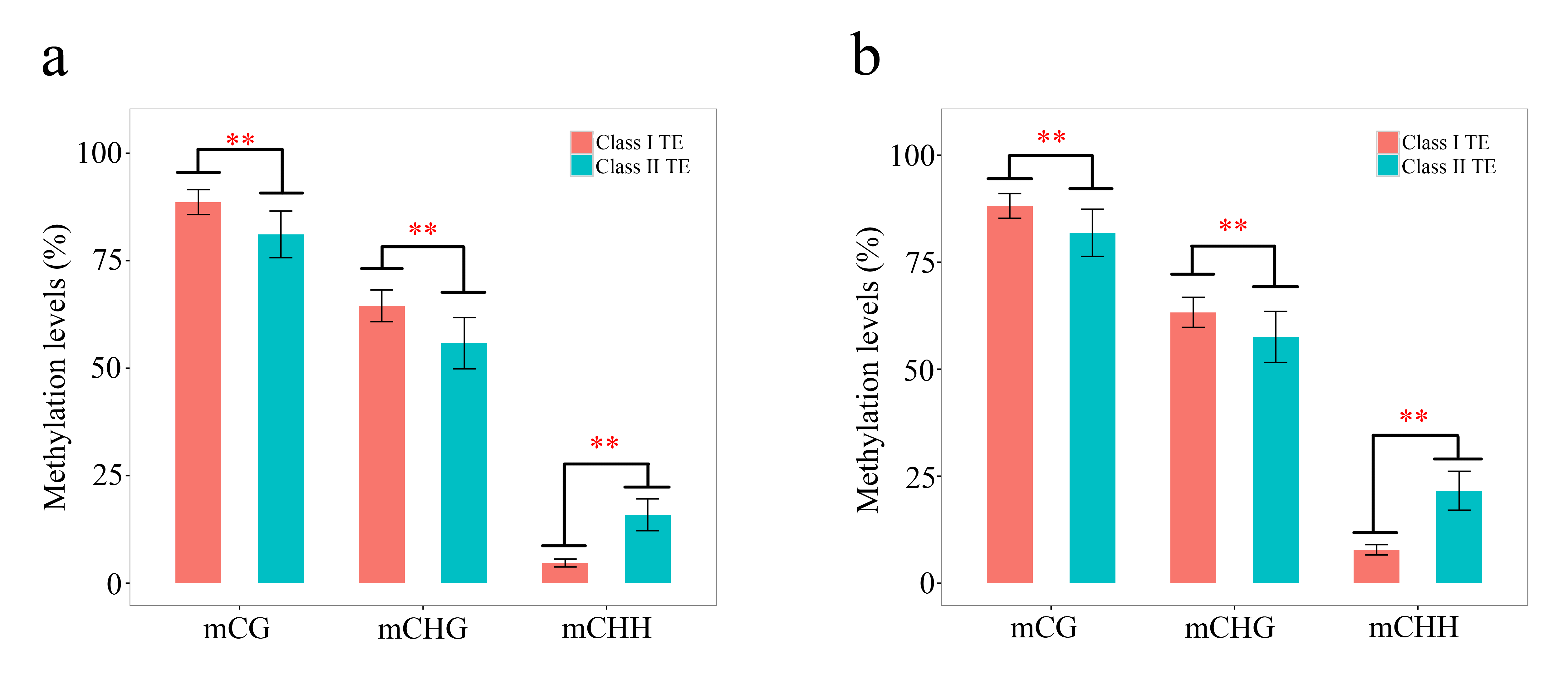

Supplement: Supplementary file 7 — Additional file 7: Figure S4. Methylation levels of Class I and Class II TEs in the leaf (a) and root (b) of switchgrass. [file 13068_2018_1202_MOESM7_ESM.tif]

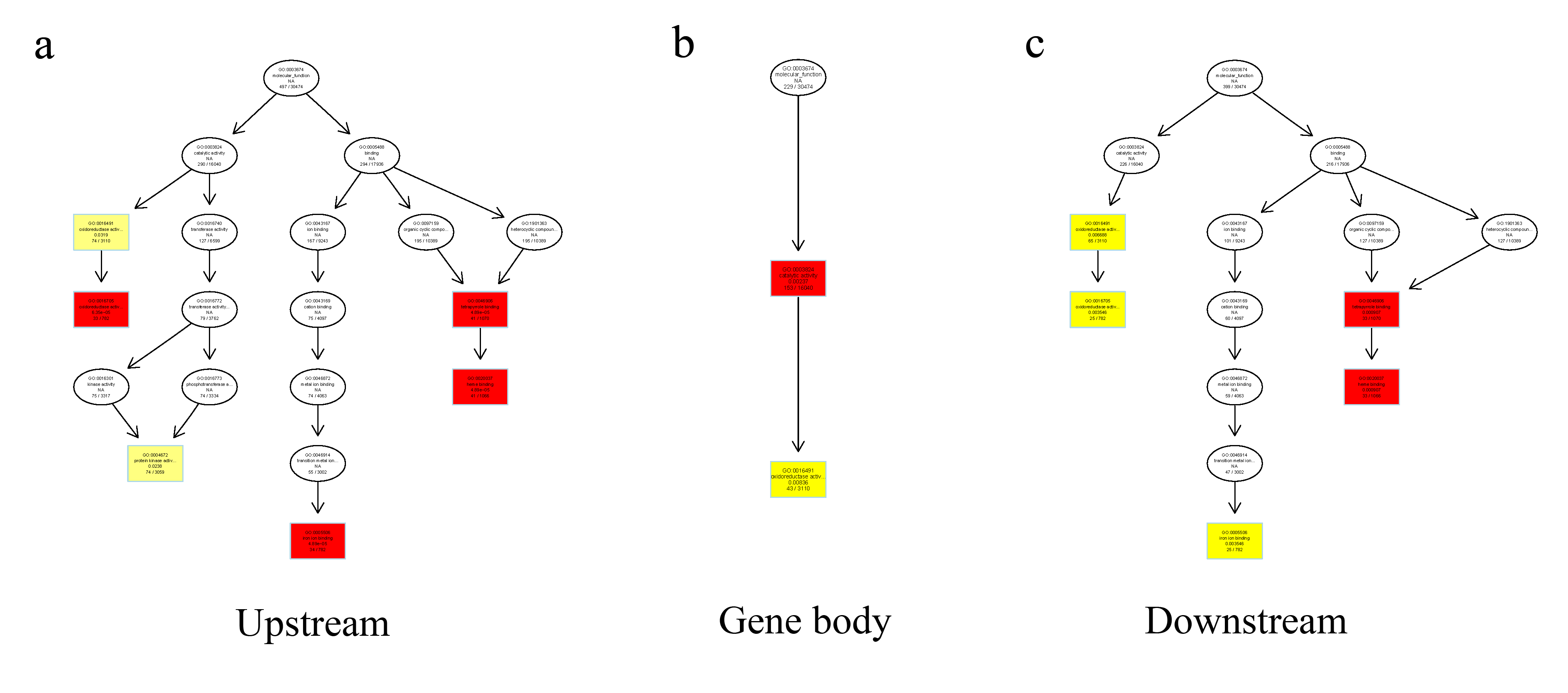

Supplement: Supplementary file 13 — Additional file 13: Figure S5. Molecular function for genes covered by the CHH DMRs in the GO annotation in gene upstream (a), body (b), and downstream (c). The colored boxes mean the adjust p < 0.05, and the more deeper color, the less adjust p values for the boxes. [file 13068_2018_1202_MOESM13_ESM.tif]

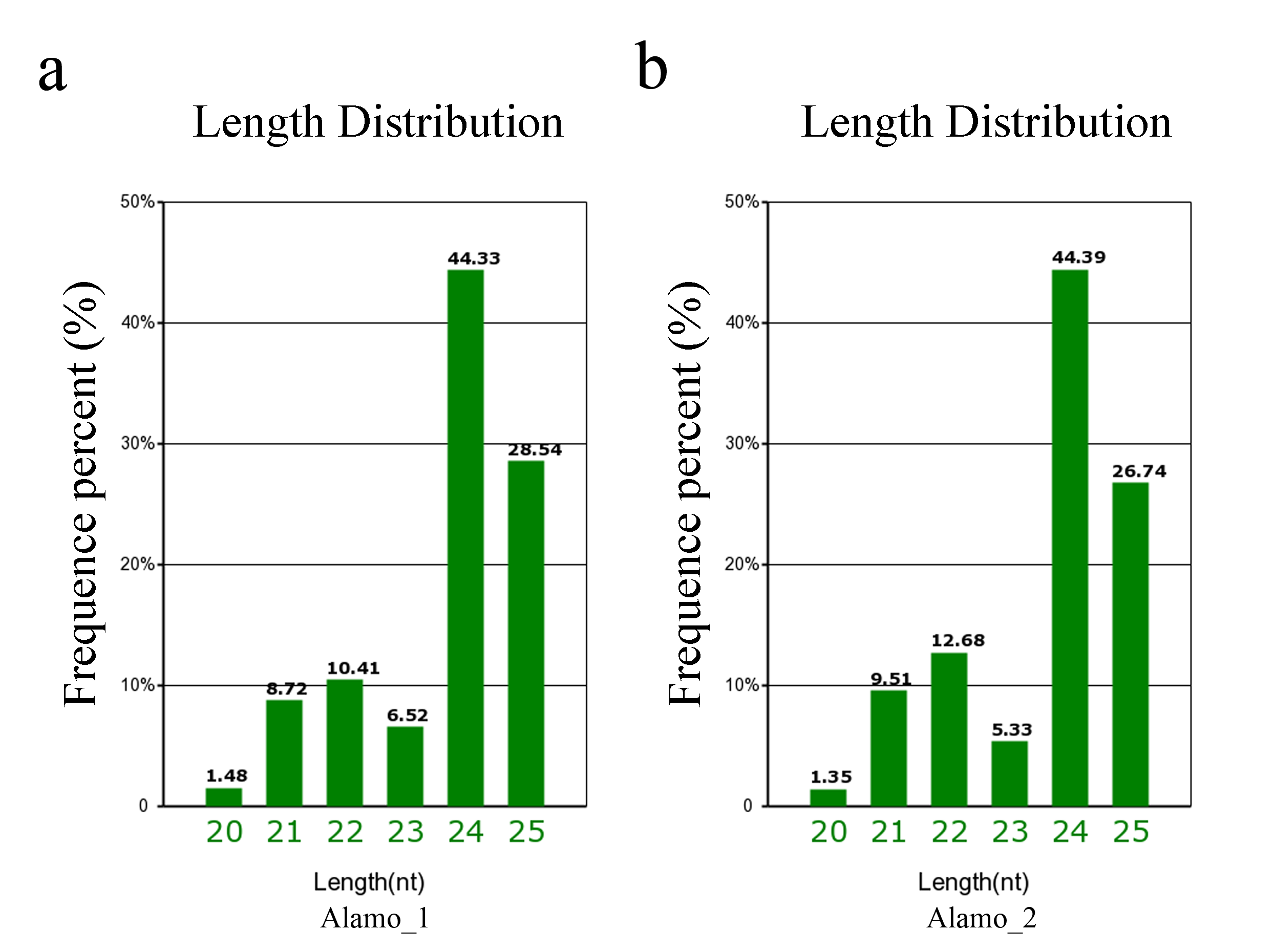

Supplement: Supplementary file 18 — Additional file 18: Table S8. Length distribution of siRNAs (from 20 to 25 nucleotides) in switchgrass for two biological repeats including Alamo_1 (a) and Alamo_2 (b). [file 13068_2018_1202_MOESM18_ESM.tif]

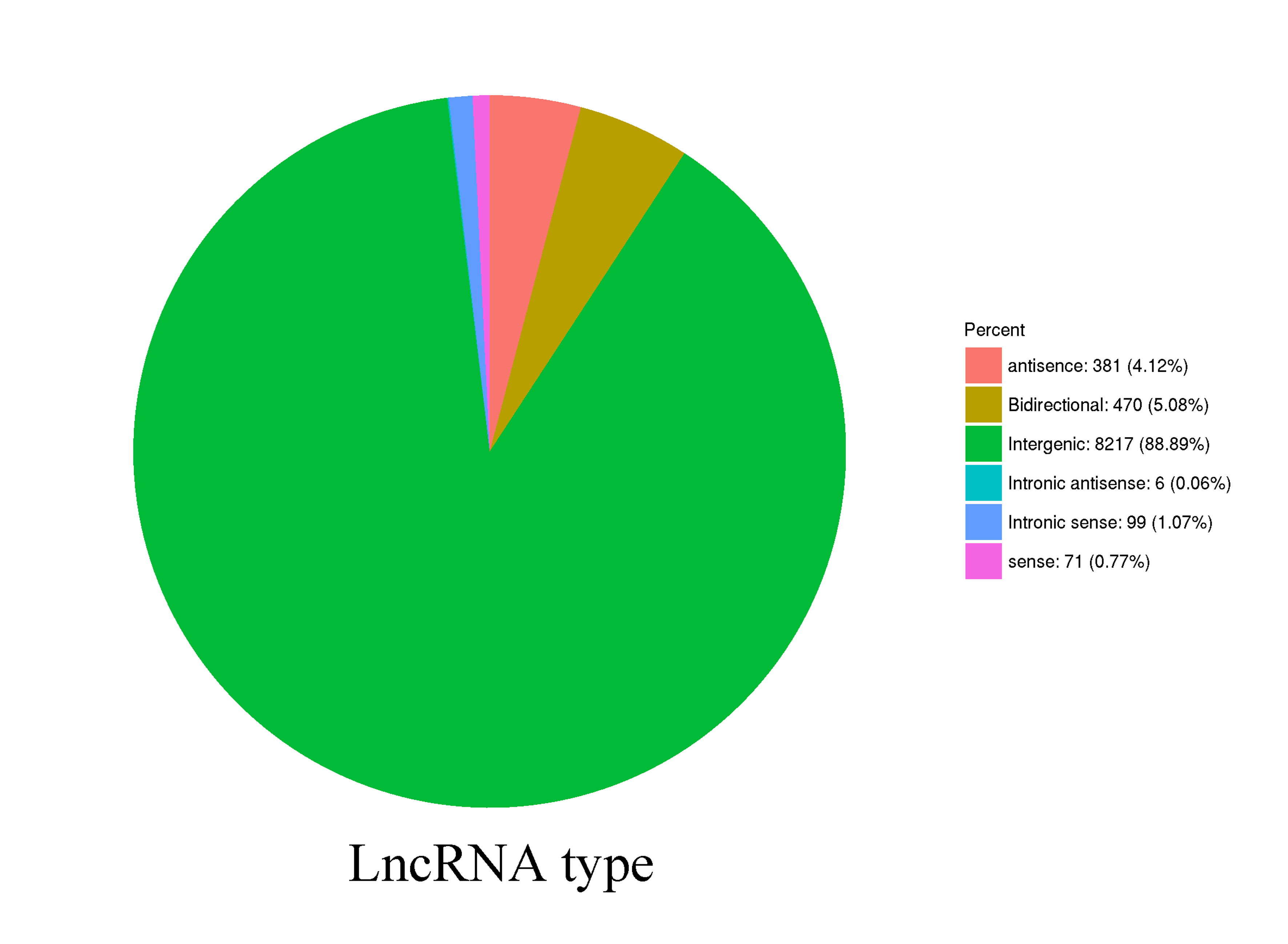

Supplement: Supplementary file 22 — Additional file 22: Figure S7. Proportion of six different types of lncRNA in switchgrass. [file 13068_2018_1202_MOESM22_ESM.tif]

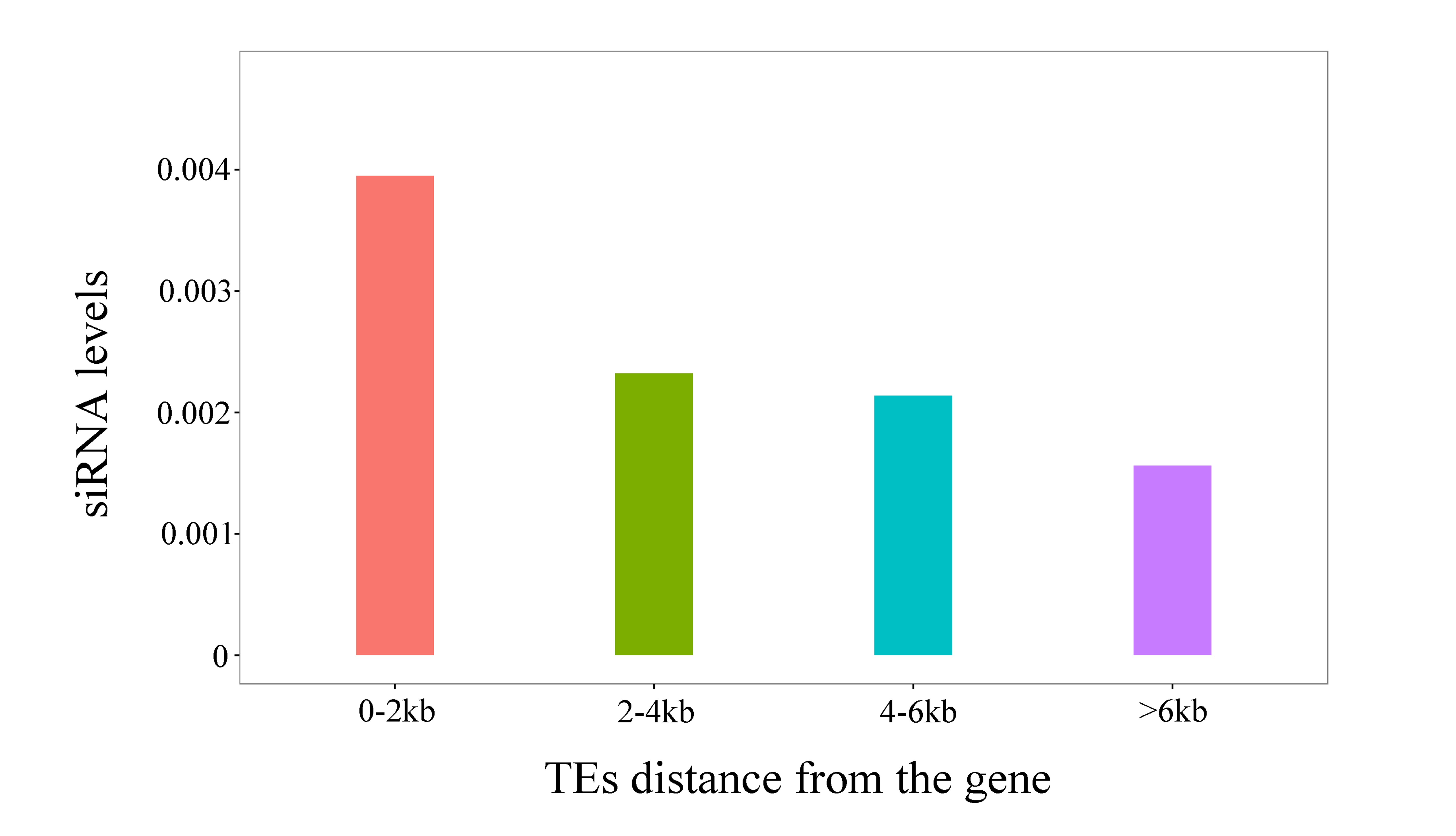

Supplement: Supplementary file 27 — Additional file 27: Figure S8. siRNA levels (per bp per million reads) in TEs relative to the distance from the nearest gene. [file 13068_2018_1202_MOESM27_ESM.tif]
