# Supplementary material for: siRNAs regulate DNA methylation and interfere with gene and lncRNA expression in the heterozygous polyploid switchgrass
Source: Biotechnol Biofuels. 2018 Jul 24;11:208. doi: 10.1186/s13068-018-1202-0 (PMC6058383; doi:10.1186/s13068-018-1202-0)
Supplement: Supplementary file 5 — Additional file 5: Table S2. The levels of mCG, mCHG, and mCHH and genome size in genomes of different species. [file 13068_2018_1202_MOESM5_ESM.docx]

**Table S2** The levels of mCG, mCHG, and mCHH and genome size in genomes of different species.

| Species | mCG (%) | mCHG (%) | mCHH (%) | Genome size (Mb) | Reference |
| --- | --- | --- | --- | --- | --- |
| *Arabidopsis thaliana* | 30.45 | 9.99 | 3.91 | 135.0 | ([Schmitz *et al.*, 2011](#_ENREF_6)) |
| *Brassica rapa* | 37.18 | 17.28 | 4.44 | 283.8 | ([Niederhuth *et al.*, 2016](#_ENREF_4)) |
| *Glycine max* | 71.07 | 45.16 | 5.09 | 978.0 | ([Schmitz *et al.*, 2013](#_ENREF_5)) |
| *Medicago truncatula* | 59.80 | 16.95 | 5.10 | 390.0 | ([Niederhuth et al., 2016](#_ENREF_4)) |
| *Populus trichocarpa* | 43.95 | 26.78 | 5.01 | 422.9 | ([Niederhuth et al., 2016](#_ENREF_4)) |
| *Zea mays* | 84.76 | 73.25 | 5.81 | 2665.0 | ([Gent *et al.*, 2013](#_ENREF_2)) |
| *Oryza sativa* | 58.37 | 30.95 | 5.52 | 372.0 | ([Hume *et al.*, 2013](#_ENREF_3)) |
| *Brachypodium distachyum* | 62.34 | 40.80 | 2.12 | 272.0 | ([Eichten *et al.*, 2016](#_ENREF_1)) |
| *Panicum virgatum* | 60.34 | 40.06 | 4.16 | 1165.7 |  |

**Reference**

Eichten SR, Stuart T, Srivastava A, Lister R, and Borevitz JO DNA methylation profiles of diverse *Brachypodium distachyon* aligns with underlying genetic diversity. Genome Res. 2016;26:1520-1531.

Gent JI, Ellis NA, Guo L, Harkess A, Yao YY, Zhang XY, and Dawe RK CHH islands: de novo DNA methylation in near-gene chromatin regulation in maize. Genome Res. 2013;23:628-637.

Hume S, Ding B, Simon SA, Feng S, Maria B, Matteo P, Wang GL, Meyers BC, and Jacobsen SE Plants regenerated from tissue culture contain stable epigenome changes in rice. Elife. 2013;2:e00354.

Niederhuth CE, Bewick AJ, Ji L, Alabady MS, Kim KD, Li Q, Rohr NA, Rambani A, Burke JM, and Udall JA Widespread natural variation of DNA methylation within angiosperms. Genome Biol. 2016;17:194.

Schmitz R, He Y, Valdés-López O, Khan S, Joshi T, Urich M, Nery J, Diers B, Xu D, Stacey G, and Ecker J Epigenome-wide inheritance of cytosine methylation variants in a recombinant inbred population. Genome Res. 2013;23:1663-1674.

Schmitz RJ, Schultz MD, Lewsey MG, O’Malley RC, Urich MA, Libiger O, Schork NJ, and Ecker JR Transgenerational epigenetic instability is a source of novel methylation variants. Sci. 2011;334:369-373.
