# Supplementary material for: siRNAs regulate DNA methylation and interfere with gene and lncRNA expression in the heterozygous polyploid switchgrass
Source: Biotechnol Biofuels. 2018 Jul 24;11:208. doi: 10.1186/s13068-018-1202-0 (PMC6058383; doi:10.1186/s13068-018-1202-0)
Supplement: Supplementary file 8 — Additional file 8: Table S3. Comparison of methylation levels between class I and class II transposons in leaf and root tissues. [file 13068_2018_1202_MOESM8_ESM.docx]

**Table S3** Comparison of methylation levels between class I and class II transposons in leaf and root tissues.

| Tissue | Context | Methylation levels (%) | | *p* value^a^ |
| --- | --- | --- | --- | --- |
|  |  | Class I | Class II |  |
| Leaf | mCG | 85.04 | 77.85 | < 2.2e-16 |
|  | mCHG | 62.78 | 54.05 | < 2.2e-16 |
|  | mCHH | 5.03 | 15.80 | < 2.2e-16 |
| Root | mCG | 85.66 | 79.84 | < 2.2e-16 |
|  | mCHG | 62.26 | 56.43 | < 2.2e-16 |
|  | mCHH | 8.14 | 21.56 | < 2.2e-16 |

Note: a: comparison of methylation levels through ANOVA test. *p* value < 0.05, means significant difference. *p* value < 0.01, means highly significant difference.
