# Supplementary material for: siRNAs regulate DNA methylation and interfere with gene and lncRNA expression in the heterozygous polyploid switchgrass
Source: Biotechnol Biofuels. 2018 Jul 24;11:208. doi: 10.1186/s13068-018-1202-0 (PMC6058383; doi:10.1186/s13068-018-1202-0)
Supplement: Supplementary file 9 — Additional file 9: Table S4. Comparison of methylation levels in genic and TE regions between switchgrass leaf and root tissues. [file 13068_2018_1202_MOESM9_ESM.docx]

**Table S4** Comparison of methylation levels in genic and TE regions between switchgrass leaf and root tissues.

| Type | Context | Position | Average methylation level (%)  in leaf | Average methylation level (%)  in root | *p* value^a^ |
| --- | --- | --- | --- | --- | --- |
| Genic region | mCG | Upstream | 0.44 | 0.38 | 0.006374 |
|  |  | Body | 0.36 | 0.32 | 0.024083 |
|  |  | Downstream | 0.40 | 0.36 | 0.001189 |
|  | mCHG | Upstream | 0.26 | 0.22 | 0.000135 |
|  |  | Body | 0.14 | 0.12 | 0.003216 |
|  |  | Downstream | 0.21 | 0.18 | 0.000865 |
|  | mCHH | Upstream | 0.06 | 0.07 | 0.006067 |
|  |  | Body | 0.03 | 0.03 | 0.000419 |
|  |  | Downstream | 0.05 | 0.05 | 2.14E-12 |
| TE region | mCG | Upstream | 0.81 | 0.80 | 0.401763 |
|  |  | Body | 0.84 | 0.84 | 0.546643 |
|  |  | Downstream | 0.79 | 0.78 | 0.369837 |
|  | mCHG | Upstream | 0.57 | 0.56 | 0.346267 |
|  |  | Body | 0.64 | 0.62 | 0.056638 |
|  |  | Downstream | 0.56 | 0.54 | 0.148894 |
|  | mCHH | Upstream | 0.04 | 0.07 | 1.000000 |
|  |  | Body | 0.04 | 0.07 | 5.1E-163 |
|  |  | Downstream | 0.04 | 0.07 | 1.19E-34 |

Note: a: comparison of methylation levels through ANOVA test. *p* value < 0.05, means significant difference. *p* value < 0.01, means highly significant difference.
