# Supplementary material for: siRNAs regulate DNA methylation and interfere with gene and lncRNA expression in the heterozygous polyploid switchgrass
Source: Biotechnol Biofuels. 2018 Jul 24;11:208. doi: 10.1186/s13068-018-1202-0 (PMC6058383; doi:10.1186/s13068-018-1202-0)
Supplement: Supplementary file 10 — Additional file 10: Table S5. Comparison of methylation levels in different TE types between switchgrass leaf and root tissues. [file 13068_2018_1202_MOESM10_ESM.docx]

**Table S5** Comparison of methylation levels in different TE types between switchgrass leaf and root tissues.

| TE Type | Context | Position | Average methylation level (%)  in leaf | Average methylation level (%)  in root | *p* value^a^ |
| --- | --- | --- | --- | --- | --- |
| Copia | mCG | Upstream | 0.76 | 0.77 | 0.7569748 |
|  |  | Body | 0.89 | 0.89 | 9.324E-14 |
|  |  | Downstream | 0.74 | 0.75 | 0.7983829 |
|  | mCHG | Upstream | 0.54 | 0.53 | 0.5924824 |
|  |  | Body | 0.67 | 0.66 | 0.0218541 |
|  |  | Downstream | 0.52 | 0.51 | 0.5385209 |
|  | mCHH | Upstream | 0.05 | 0.07 | 1.514E-57 |
|  |  | Body | 0.05 | 0.09 | 3.216E-63 |
|  |  | Downstream | 0.04 | 0.07 | 8.077E-49 |
| Gypsy | mCG | Upstream | 0.82 | 0.82 | 0.6878237 |
|  |  | Body | 0.89 | 0.89 | 4.449E-08 |
|  |  | Downstream | 0.80 | 0.81 | 0.7218451 |
|  | mCHG | Upstream | 0.60 | 0.59 | 0.164831 |
|  |  | Body | 0.70 | 0.68 | 8.161E-09 |
|  |  | Downstream | 0.60 | 0.59 | 0.1579212 |
|  | mCHH | Upstream | 0.04 | 0.07 | 1.382E-90 |
|  |  | Body | 0.04 | 0.07 | 2.521E-69 |
|  |  | Downstream | 0.04 | 0.07 | 6.396E-68 |
| LTR-Other | mCG | Upstream | 0.62 | 0.62 | 0.5348803 |
|  |  | Body | 0.85 | 0.85 | 0.8946232 |
|  |  | Downstream | 0.62 | 0.61 | 0.4902904 |
|  | mCHG | Upstream | 0.40 | 0.39 | 0.3561242 |
|  |  | Body | 0.59 | 0.58 | 0.4589623 |
|  |  | Downstream | 0.40 | 0.39 | 0.2591275 |
|  | mCHH | Upstream | 0.04 | 0.07 | 7.834E-18 |
|  |  | Body | 0.09 | 0.13 | 4.386E-15 |
|  |  | Downstream | 0.04 | 0.07 | 3.005E-22 |
| LINE | mCG | Upstream | 0.54 | 0.54 | 0.0153612 |
|  |  | Body | 0.71 | 0.70 | 0.3726903 |
|  |  | Downstream | 0.52 | 0.52 | 0.0130829 |
|  | mCHG | Upstream | 0.30 | 0.29 | 0.0008056 |
|  |  | Body | 0.45 | 0.44 | 0.0032815 |
|  |  | Downstream | 0.28 | 0.28 | 0.1677391 |
|  | mCHH | Upstream | 0.04 | 0.05 | 1.085E-67 |
|  |  | Body | 0.04 | 0.06 | 6.284E-42 |
|  |  | Downstream | 0.04 | 0.05 | 1.238E-54 |
| SINE | mCG | Upstream | 0.53 | 0.53 | 0.8287993 |
|  |  | Body | 0.83 | 0.84 | 0.5251535 |
|  |  | Downstream | 0.55 | 0.55 | 0.9262316 |
|  | mCHG | Upstream | 0.32 | 0.32 | 0.9307919 |
|  |  | Body | 0.60 | 0.61 | 0.8927421 |
|  |  | Downstream | 0.35 | 0.35 | 0.9342469 |
|  | mCHH | Upstream | 0.05 | 0.07 | 1.742E-20 |
|  |  | Body | 0.07 | 0.12 | 4.12E-15 |
|  |  | Downstream | 0.05 | 0.08 | 1.698E-16 |
| DNA-Other | mCG | Upstream | 0.41 | 0.40 | 0.3186607 |
|  |  | Body | 0.78 | 0.81 | 0.0030706 |
|  |  | Downstream | 0.41 | 0.40 | 0.1839996 |
|  | mCHG | Upstream | 0.26 | 0.25 | 0.3986551 |
|  |  | Body | 0.61 | 0.64 | 0.0939602 |
|  |  | Downstream | 0.26 | 0.25 | 0.2426912 |
|  | mCHH | Upstream | 0.06 | 0.08 | 3.476E-06 |
|  |  | Body | 0.30 | 0.41 | 3.071E-17 |
|  |  | Downstream | 0.06 | 0.08 | 7.163E-09 |
| hAT | mCG | Upstream | 0.52 | 0.51 | 0.2843537 |
|  |  | Body | 0.83 | 0.83 | 0.1948183 |
|  |  | Downstream | 0.52 | 0.51 | 0.2826515 |
|  | mCHG | Upstream | 0.32 | 0.31 | 0.3299229 |
|  |  | Body | 0.49 | 0.50 | 0.1155802 |
|  |  | Downstream | 0.32 | 0.31 | 0.3047229 |
|  | mCHH | Upstream | 0.05 | 0.07 | 2.671E-18 |
|  |  | Body | 0.10 | 0.13 | 1.901E-22 |
|  |  | Downstream | 0.05 | 0.07 | 9.61E-19 |
| MULE-MuDR | mCG | Upstream | 0.57 | 0.56 | 0.5769647 |
|  |  | Body | 0.84 | 0.85 | 0.0002578 |
|  |  | Downstream | 0.58 | 0.57 | 0.4248912 |
|  | mCHG | Upstream | 0.38 | 0.38 | 0.4886548 |
|  |  | Body | 0.66 | 0.66 | 0.5698749 |
|  |  | Downstream | 0.39 | 0.38 | 0.3564224 |
|  | mCHH | Upstream | 0.05 | 0.07 | 4.258E-24 |
|  |  | Body | 0.09 | 0.13 | 9.72E-27 |
|  |  | Downstream | 0.05 | 0.07 | 2.226E-31 |
| EnSpm | mCG | Upstream | 0.61 | 0.60 | 0.511658 |
|  |  | Body | 0.86 | 0.86 | 0.1012361 |
|  |  | Downstream | 0.59 | 0.59 | 0.4489362 |
|  | mCHG | Upstream | 0.41 | 0.40 | 0.740117 |
|  |  | Body | 0.60 | 0.61 | 0.0805807 |
|  |  | Downstream | 0.39 | 0.39 | 0.9166526 |
|  | mCHH | Upstream | 0.05 | 0.07 | 3.484E-17 |
|  |  | Body | 0.11 | 0.14 | 8.574E-25 |
|  |  | Downstream | 0.05 | 0.07 | 1.639E-18 |
| Stowaway | mCG | Upstream | 0.39 | 0.39 | 0.2879146 |
|  |  | Body | 0.67 | 0.70 | 0.0002799 |
|  |  | Downstream | 0.40 | 0.39 | 0.2568919 |
|  | mCHG | Upstream | 0.20 | 0.20 | 0.8846443 |
|  |  | Body | 0.38 | 0.42 | 1.589E-05 |
|  |  | Downstream | 0.21 | 0.21 | 0.9241038 |
|  | mCHH | Upstream | 0.04 | 0.06 | 9.532E-31 |
|  |  | Body | 0.15 | 0.23 | 3.221E-17 |
|  |  | Downstream | 0.04 | 0.06 | 1.431E-46 |

Note: a: comparison of methylation levels between leaf and root tissues by using ANOVA test. *p* value < 0.05, means significant difference. *p* value < 0.01, means highly significant difference.
