# Supplementary material for: siRNAs regulate DNA methylation and interfere with gene and lncRNA expression in the heterozygous polyploid switchgrass
Source: Biotechnol Biofuels. 2018 Jul 24;11:208. doi: 10.1186/s13068-018-1202-0 (PMC6058383; doi:10.1186/s13068-018-1202-0)
Supplement: Supplementary file 15 — Additional file 15: Table S6. The number of mRNA and lncRNA in the four expression levels. [file 13068_2018_1202_MOESM15_ESM.docx]

**Table S6** The number of mRNA and lncRNA in the four expression levels.

| Sample | The number mRNA and lncRNA of four expression levels | | | |
| --- | --- | --- | --- | --- |
|  | None^a^ | Low^b^ | Middle^c^ | High^d^ |
| mRNA | 77165 | 24460 | 24460 | 24460 |
| LncRNA | 5267 | 1325 | 1326 | 1326 |

**Note:** a: non-expressed group, reads per kilobase per million reads mapped [RPKM] ≤ 1. b: low-expressed group, reads per kilobase per million reads mapped (1 < RPKM ≤ 10). c: middle-expressed group, reads per kilobase per million reads mapped (10 < RPKM ≤ 100). d: high-expressed group, reads per kilobase per million reads mapped (RPKM > 100).
