# Supplementary material for: siRNAs regulate DNA methylation and interfere with gene and lncRNA expression in the heterozygous polyploid switchgrass
Source: Biotechnol Biofuels. 2018 Jul 24;11:208. doi: 10.1186/s13068-018-1202-0 (PMC6058383; doi:10.1186/s13068-018-1202-0)
Supplement: Supplementary file 16 — Additional file 16: Table S7. Correlation analysis between methylation levels and gene expression in different genic regions. [file 13068_2018_1202_MOESM16_ESM.docx]

**Table S7** Correlation analysis between methylation levels and gene expression in different genic regions.

| Methylated contexts | Regions | *p* value^a^ | *rho* | Strength of the correlation | Positively (+) or negatively (-) |
| --- | --- | --- | --- | --- | --- |
| mCG | Upstream | 2.42E-51 | -0.080 | very weak^b^ | - |
|  | Body | 2.25E-267 | 0.279 | weak^c^ | + |
|  | Downstream | 2.02E-289 | -0.191 | very weak | - |
|  |  |  |  |  |  |
| mCHG | Upstream | 5.25E-07 | -0.027 | very weak | - |
|  | Body | 1.14E-17 | -0.045 | very weak | - |
|  | Downstream | 2.71E-311 | -0.198 | very weak | - |
|  |  |  |  |  |  |
| mCHH | Upstream | 5.05E-47 | 0.078 | very weak | + |
|  | Body | 7.6E-39 | -0.068 | very weak | - |
|  | Downstream | 6.84E-92 | -0.107 | very weak | - |

**Note:** a: *p* value < 0.05 means significant correlation; b: the absolute value of *rho* < 0.2. c: 0.2 ≤ the absolute value of *rho* < 0.4.
