# Supplementary material for: siRNAs regulate DNA methylation and interfere with gene and lncRNA expression in the heterozygous polyploid switchgrass
Source: Biotechnol Biofuels. 2018 Jul 24;11:208. doi: 10.1186/s13068-018-1202-0 (PMC6058383; doi:10.1186/s13068-018-1202-0)
Supplement: Supplementary file 17 — Additional file 17: Table S8. Regression analysis and 10-fold cross-validation between DNA methylation and gene expression based on zero hurdle model. [file 13068_2018_1202_MOESM17_ESM.docx]

**Table S8** Regression analysis and 10-fold cross-validation between DNA methylation and gene expression based on zero hurdle model.

| Context | Position | Model coefficient ^a^ | *p* value ^b^ | Q^2 c^ |
| --- | --- | --- | --- | --- |
| mCG | Upstream | -0.00535 | 4.75E-48 | 0.001807 |
|  | Body | 0.01255 | 2.83E-244 | 0.000288 |
|  | Downstream | -0.01306 | 2.82E-263 | 0.00244 |
| mCHG | Upstream | -0.0019 | 4.68E-05 | 0.000388 |
|  | Body | -0.03048 | 6.41E-246 | 0.005059 |
|  | Downstream | -0.01728 | 1.42E-231 | 0.001942 |
| mCHH | Upstream | 0.02431 | 1.63E-41 | 0.000151 |
|  | Body | -0.14315 | 1.98E-133 | 0.003575 |
|  | Downstream | -0.02264 | 4.96E-22 | 0.000139 |

Note: a: model coefficient > 0, means positive correlation; model coefficient < 0, means negative correlation. b: *p* value < 0.05, means significance of the correlation. c: 10-fold cross-validate value calculated by a formula, Q2 = 1 – PRESS (Predictive Error Sum of Squares)/TSS (Total Sum of Squares).
