# Supplementary material for: siRNAs regulate DNA methylation and interfere with gene and lncRNA expression in the heterozygous polyploid switchgrass
Source: Biotechnol Biofuels. 2018 Jul 24;11:208. doi: 10.1186/s13068-018-1202-0 (PMC6058383; doi:10.1186/s13068-018-1202-0)
Supplement: Supplementary file 19 — Additional file 19: Table S9. Comparison of methylation levels between siRNA uniquely mapping region and without that region. [file 13068_2018_1202_MOESM19_ESM.docx]

**Table S9** Comparison of methylation levels between siRNA uniquely mapping region and without that region.

| Context | Methylation levels (%) | | *p* value^a^ |
| --- | --- | --- | --- |
|  | siRNA mapping | no siRNA mapping |  |
| mCG | 71.95 | 64.85 | < 2.2e-16 |
| mCHG | 52.52 | 40.83 | < 2.2e-16 |
| mCHH | 14.28 | 4.14 | < 2.2e-16 |

Note: a: comparison of methylation levels through Fisher’s exact test. *p* value < 0.05, means significant difference. *p* value < 0.01, means highly significant difference.
