# Supplementary material for: siRNAs regulate DNA methylation and interfere with gene and lncRNA expression in the heterozygous polyploid switchgrass
Source: Biotechnol Biofuels. 2018 Jul 24;11:208. doi: 10.1186/s13068-018-1202-0 (PMC6058383; doi:10.1186/s13068-018-1202-0)
Supplement: Supplementary file 20 — Additional file 20: Table S10. The correlation analysis between gene methylation and siRNA expression. [file 13068_2018_1202_MOESM20_ESM.docx]

**Table S10 The correlation analysis between gene methylation and siRNA expression.**

| Context | Region | Expression | | | |
| --- | --- | --- | --- | --- | --- |
|  |  | *p* value^a^ | *rho* | Strength of the correlation | Positively (+) or negatively (-) |
| mCG | Upstream | 2.77E-46 | -0.085 | Very weak^b^ | - |
|  | Body | 1.68E-07 | 0.044 | Very weak | + |
|  | Downstream | 3.26E-192 | -0.184 | Very weak | - |
|  |  |  |  |  |  |
| mCHG | Upstream | 0.103 | 0.010 | None^c^ | None |
|  | Body | 1.39E-110 | 0.185 | Very weak | + |
|  | Downstream | 2.56E-08 | -0.035 | Very weak | - |
|  |  |  |  |  |  |
| mCHH | Upstream | 1.02E-171 | 0.253 | Weak^d^ | + |
|  | Body | 3.42E-214 | 0.228 | Weak | + |
|  | Downstream | 2.02E-115 | 0.317 | Weak | + |

**Note:** a: *p* value < 0.05 means significant correlation; b: the absolute value of *rho* < 0.2; c: no correlation (*p* value > 0.05); d: 0.2 ≤ the absolute value of *rho* < 0.4.
