# Supplementary material for: siRNAs regulate DNA methylation and interfere with gene and lncRNA expression in the heterozygous polyploid switchgrass
Source: Biotechnol Biofuels. 2018 Jul 24;11:208. doi: 10.1186/s13068-018-1202-0 (PMC6058383; doi:10.1186/s13068-018-1202-0)
Supplement: Supplementary file 21 — Additional file 21: Table S11. Regression analysis and 10-fold cross-validation between DNA methylation and siRNA expression based on negative binomial model. [file 13068_2018_1202_MOESM21_ESM.docx]

**Table S11** Regression analysis and 10-fold cross-validation between DNA methylation and siRNA expression based on negative binomial model.

| Context | Position | Model coefficient^a^ | *p* value^b^ | Q^2 c^ |
| --- | --- | --- | --- | --- |
| mCG | Upstream | 0.00177 | 6.52E-11 | -0.001216 |
|  | Body | 0.00845 | 1.84E-69 | 0.001346 |
|  | Downstream | -0.00412 | 1.27E-46 | 0.000395 |
| mCHG | Upstream | 0.00558 | 2.07E-60 | 0.000280 |
|  | Body | 0.00835 | 1.98E-33 | 0.001228 |
|  | Downstream | -0.00015 | 0.69666 | -0.001111 |
| mCHH | Upstream | 0.04113 | 3.47E-246 | 0.002506 |
|  | Body | 0.03535 | 4.42E-27 | 0.001774 |
|  | Downstream | 0.02015 | 5.83E-34 | 0.001528 |

Note: a: model coefficient > 0, means positive correlation; model coefficient < 0, means negative correlation. b: *p* value < 0.05, means significance of the correlation. c: 10-fold cross-validate value calculated by a formula, Q2 = 1 – PRESS (Predictive Error Sum of Squares)/TSS (Total Sum of Squares).
