# Supplementary material for: siRNAs regulate DNA methylation and interfere with gene and lncRNA expression in the heterozygous polyploid switchgrass
Source: Biotechnol Biofuels. 2018 Jul 24;11:208. doi: 10.1186/s13068-018-1202-0 (PMC6058383; doi:10.1186/s13068-018-1202-0)
Supplement: Supplementary file 23 — Additional file 23: Table S12. Prediction of lncRNA families in switchgrass. [file 13068_2018_1202_MOESM23_ESM.docx]

**Table S12** Prediction of lncRNA families in switchgrass.

| **Family_Name** | **Full name** | **Function** | **Family_Accession** | **LncRNA** | **start** | **end** | **E-value** | **Score** | **GC%** |
| --- | --- | --- | --- | --- | --- | --- | --- | --- | --- |
| HAR1A | Human accelerated region 1 A | Schizophrenia (Tolosa et al. 2008) | RF00635 | TCONS_00013515 | 335 | 443 | 0.0023 | 26.6 | 0.44 |
| Xist_exon1 | X-inactive specific transcript exon 1 | The major effector of the X inactivation process (Herzing et al. 1997) | RF01880 | TCONS_00106038 | 194 | 111 | 0.0073 | 22.8 | 0.42 |
| SOX2OT_exon2 | SOX2 overlapping transcript exon 2 | The important regulator of neurogenesis (Fantes et al. 2003) | RF01952 | TCONS_00060457 | 196 | 256 | 0.0014 | 26 | 0.43 |
| H19_3 | H19 conserved region 3 | Negative regulation (or limiting) of body weight and cell proliferation (Gabory et al. 2009) | RF01974 | TCONS_00165105 | 464 | 560 | 0.002 | 24.8 | 0.38 |
| ZEB2_AS1_2 | ZEB2 antisense RNA 1 conserved region 2 | Tumor growth and metastasis in hepatocellular carcinoma (Lan et al. 2016) | RF01985 | TCONS_00061590 | 259 | 298 | 0.0028 | 28.8 | 0.6 |
| RFPL3 | Ret Finger Protein-Like genes 3 | promote lung cancer growth (Qin et al. 2015) | RF02171 | TCONS_00019556 | 127 | 43 | 6.40E-05 | 29.8 | 0.58 |
| RFPL3 | Ret Finger Protein-Like genes | promote lung cancer growth (Qin et al. 2015) | RF02171 | TCONS_00086003 | 109 | 32 | 0.0017 | 24.8 | 0.41 |
