# Supplementary material for: siRNAs regulate DNA methylation and interfere with gene and lncRNA expression in the heterozygous polyploid switchgrass
Source: Biotechnol Biofuels. 2018 Jul 24;11:208. doi: 10.1186/s13068-018-1202-0 (PMC6058383; doi:10.1186/s13068-018-1202-0)
Supplement: Supplementary file 24 — Additional file 24: Table S13. Annotation of targets of miR169 and miR171. [file 13068_2018_1202_MOESM24_ESM.docx]

**Table S13** Annotation of targets of MIR169 and MIR171.

| miRNA | Targets of miRNA | Description |
| --- | --- | --- |
| MIR169 | Pavir.1KG321800.1 | pectinesterase inhibitor domain containing protein, putative, expressed |
|  | Pavir.2NG152900.1 | pectinesterase inhibitor domain containing protein, putative, expressed |
|  | Pavir.3KG498900.1 | mitochondrial import inner membrane translocase subunit Tim, putative, expressed |
|  | Pavir.4NG238700.1 | OsSub51 - Putative Subtilisin homologue, expressed |
|  | Pavir.7NG105500.1 | stress responsive protein, putative, expressed |
|  | Pavir.7NG105500.2 | stress responsive protein, putative, expressed |
|  | Pavir.9NG369800.1 | transposon protein, putative, CACTA, En/Spm sub-class, expressed |
|  | Pavir.J271100.1 | pectinesterase inhibitor domain containing protein, putative, expressed |
|  |  |  |
| MIR171 | Pavir.1KG431100.1 | scarecrow, putative, expressed |
|  | Pavir.1KG431100.2 | scarecrow, putative, expressed |
|  | Pavir.1KG545200.1 | tetratricopeptide-like helical, putative, expressed |
|  | Pavir.1NG288600.1 | SacI homology domain containing protein, expressed |
|  | Pavir.1NG288600.2 | SacI homology domain containing protein, expressed |
|  | Pavir.1NG288600.3 | SacI homology domain containing protein, expressed |
|  | Pavir.1NG556600.1 | tetratricopeptide-like helical, putative, expressed |
|  | Pavir.4KG010200.1 | scarecrow, putative, expressed |
|  | Pavir.4NG003100.1 | scarecrow, putative, expressed |
|  | Pavir.5KG290700.1 | retrotransposon protein, putative, Ty1-copia subclass, expressed |
|  | Pavir.5KG626400.2 | oxidoreductase, aldo/keto reductase family protein, putative, expressed |
|  | Pavir.7KG283500.1 | scarecrow, putative, expressed |
|  | Pavir.7KG283500.2 | scarecrow, putative, expressed |
|  | Pavir.7NG276500.1 | scarecrow, putative, expressed |
|  | Pavir.9KG317700.1 | scarecrow, putative, expressed |
|  | Pavir.9KG504600.1 | nodulation-signaling pathway 2 protein, putative, expressed |
|  | Pavir.9KG512500.1 | nodulation-signaling pathway 2 protein, putative, expressed |
|  | Pavir.9NG128800.1 | retrotransposon protein, putative, Ty1-copia subclass, expressed |
|  | Pavir.9NG478100.1 | scarecrow, putative, expressed |
|  | Pavir.9NG665600.1 | nodulation-signaling pathway 2 protein, putative, expressed |
|  | Pavir.9NG840200.1 | SacI homology domain containing protein, expressed |
