# Supplementary material for: siRNAs regulate DNA methylation and interfere with gene and lncRNA expression in the heterozygous polyploid switchgrass
Source: Biotechnol Biofuels. 2018 Jul 24;11:208. doi: 10.1186/s13068-018-1202-0 (PMC6058383; doi:10.1186/s13068-018-1202-0)
Supplement: Supplementary file 25 — Additional file 25: Table S14. The correlation analysis between methylation levels and lncRNA expression. [file 13068_2018_1202_MOESM25_ESM.docx]

**Table S14** The correlation analysis between methylation levels and lncRNA expression.

| Methylated context | Region | *p* value^a^ | *rho* | Strength of the correlation | Positively (+) or negatively (-) |
| --- | --- | --- | --- | --- | --- |
| mCG | Upstream | 2.11E-30 | -0.160 | Very weak^b^ | - |
|  | Body | 1.72E-41 | -0.188 | Very weak | - |
|  | Downstream | 1.81E-28 | -0.153 | Very weak | - |
|  |  |  |  |  |  |
| mCHG | Upstream | 1.70E-37 | -0.180 | Very weak | - |
|  | Body | 1.17E-31 | -0.169 | Very weak | - |
|  | Downstream | 1.61E-40 | -0.186 | Very weak | - |
|  |  |  |  |  |  |
| mCHH | Upstream | 1.30E-17 | -0.117 | Very weak | - |
|  | Body | 3.80E-12 | -0.095 | Very weak | - |
|  | Downstream | 8.28E-05 | -0.056 | Very weak | - |

**Note:** a: *p* value < 0.05 means significant correlation; b: the absolute value of *rho* < 0.2.
