# Supplementary material for: siRNAs regulate DNA methylation and interfere with gene and lncRNA expression in the heterozygous polyploid switchgrass
Source: Biotechnol Biofuels. 2018 Jul 24;11:208. doi: 10.1186/s13068-018-1202-0 (PMC6058383; doi:10.1186/s13068-018-1202-0)
Supplement: Supplementary file 26 — Additional file 26: Table S15. Regression analysis and 10-fold cross-validation between DNA methylation and lncRNA expression based on zero hurdle model. [file 13068_2018_1202_MOESM26_ESM.docx]

**Table S15** Regression analysis and 10-fold cross-validation between DNA methylation and lncRNA expression based on zero hurdle model.

| Context | Position | Model coefficient^a^ | *p* value^b^ | Q^2 c^ |
| --- | --- | --- | --- | --- |
| mCG | Upstream | -0.04453 | 5.17E-23 | 0.009029 |
|  | Body | -0.04296 | 3.50E-27 | 0.001278 |
|  | Downstream | -0.02817 | 3.38E-09 | 0.001422 |
| mCHG | Upstream | -0.04448 | 5.58E-20 | 0.000317 |
|  | Body | -0.05594 | 3.99E-16 | 0.012784 |
|  | Downstream | -0.05326 | 7.14E-23 | 0.001770 |
| mCHH | Upstream | -0.05974 | 5.15E-13 | 0.001136 |
|  | Body | -0.05545 | 7.57E-12 | 0.000337 |
|  | Downstream | -0.03096 | 0.15908 | -0.002135 |

Note: a: model coefficient > 0, means positive correlation; model coefficient < 0, means negative correlation. b: *p* value < 0.05, means significance of the correlation. c: 10-fold cross-validate value calculated by a formula, Q2 = 1 – PRESS (Predictive Error Sum of Squares)/TSS (Total Sum of Squares).
